# Detection of Fall Risk in Multiple Sclerosis by Gait Analysis—An Innovative Approach Using Feature Selection Ensemble and Machine Learning Algorithms

**DOI:** 10.3390/brainsci12111477

**Published:** 2022-10-31

**Authors:** Paula Schumann, Maria Scholz, Katrin Trentzsch, Thurid Jochim, Grzegorz Śliwiński, Hagen Malberg, Tjalf Ziemssen

**Affiliations:** 1 Institute of Biomedical Engineering, TU Dresden, Fetscherstr. 29, 01307 Dresden, Germany; 2 Center of Clinical Neuroscience, Neurological Clinic, University Hospital Carl Gustav Carus, TU Dresden, Fetscherstr. 74, 01307 Dresden, Germany

**Keywords:** Multiple Sclerosis, fall risk, gait, machine learning, feature selection ensemble

## Abstract

One of the common causes of falls in people with Multiple Sclerosis (pwMS) is walking impairment. Therefore, assessment of gait is of importance in MS. Gait analysis and fall detection can take place in the clinical context using a wide variety of available methods. However, combining these methods while using machine learning algorithms for detecting falls has not been performed. Our objective was to determine the most relevant method for determining fall risk by analyzing eleven different gait data sets with machine learning algorithms. In addition, we examined the most important features of fall detection. A new feature selection ensemble (FS-Ensemble) and four classification models (Gaussian Naive Bayes, Decision Tree, k-Nearest Neighbor, Support Vector Machine) were used. The FS-Ensemble consisted of four filter methods: Chi-square test, information gain, Minimum Redundancy Maximum Relevance and RelieF. Various thresholds (50%, 25% and 10%) and combination methods (Union, Union 2, Union 3 and Intersection) were examined. Patient-reported outcomes using specialized walking questionnaires such as the 12-item Multiple Sclerosis Walking Scale (MSWS-12) and the Early Mobility Impairment Questionnaire (EMIQ) achieved the best performances with an F1 score of 0.54 for detecting falls. A combination of selected features of MSWS-12 and EMIQ, including the estimation of walking, running and stair climbing ability, the subjective effort as well as necessary concentration and walking fluency during walking, the frequency of stumbling and the indication of avoidance of social activity achieved the best recall of 75%. The Gaussian Naive Bayes was the best classification model for detecting falls with almost all data sets. FS-Ensemble improved the classification models and is an appropriate technique for reducing data sets with a large number of features. Future research on other risk factors, such as fear of falling, could provide further insights.

## 1. Introduction

People with Multiple Sclerosis (pwMS) are often at a high risk of falling due to many neurological abnormalities associated with the disease progression [1]. There are several risk factors in pwMS for falling, such as a high Expanded Disability Status Scale (EDSS), disease activity, sensory or cognitive impairments, impaired perception resulting from visual impairment and bladder incontinence as well as gait abnormalities and fear of falling [2,3,4,5,6,7,8,9,10]. Among these risk factors, gait abnormalities are considered to be one of the most common impairments in pwMS [11,12].

There are different possibilities to assess gait impairment, for example, rater-based, sensor-based and patient-based methods. Rater-based methods are objective and give an overview of different functional areas. Sensor-based methods are standardized non-human-dependent measures that allow multidimensional viewing of gait. Patient self-assessment plays an important role in measuring gait changes in daily life and, therefore, better reflects the health status [13]. Procedures applying cognitive tasks during walking are able to detect more sensitive gait abnormalities since patients with increased cognitive impairment need more attention while walking in order to compensate for further disorders [14]. Methods that measure balance, as well as methods for early identification of mobility impairments, already reveal changes that are not yet evident in normal walking [15,16,17].

Gait parameters can be correlated with falls. A review reported that pwMS who fall have a higher overall disability stage, a worse balance compared to non-fallers, a lower walking speed and a shorter stride length [8]. Early detection of functional decline could therefore prevent falls and injuries. Most previous studies used statistical hypothesis testing to identify patients at risk of falling. Retrospective fall status classification commonly helps to determine outcomes that are sufficiently sensitive to distinguish people who have fallen from those who have not [18]. However, due to the large number of outcome variables, it is difficult to compare results across investigations. 

Machine learning algorithms allow for discovering new patterns that were hidden beneath the complexity of the data. Several studies have retrospectively analyzed fall detection with machine learning with a focus on the elderly population [19,20,21,22,23,24,25,26] and people with neurological disorders [27,28,29]. Different conventional algorithms were trained in these studies, among which Naive Bayes, Support Vector Machines, Decision Tree and k-Nearest Neighbor achieved high performances [19,20,21,22,24,25,27]. However, most of the studies considered only wearable sensor-based parameters or used a small cohort of patients. Deep learning methods applied in two further studies outperformed conventional machine learning algorithms, on the contrary [23,28], but need thousands of samples to analyze efficiently [30,31]. Research with patient-reported outcomes (PRO) and combining different assessment systems is still lacking. 

Even a small amount of noisy data can further hide patterns from machine learning algorithms. To boost the detectability, feature selection is one of the most important processing steps [19,21,26,32]. Several techniques are available, each one with its own strengths and weaknesses. Because no best method has been generalized so far [33,34,35], feature selection ensembles have been applied [36].

In our study, we incorporate different sensor methods, clinically accepted patient-reported measures and neurologist-administered assessments for detecting fallers using machine learning-based techniques. The aim of our work is to identify the most suitable methods for fall detection. We investigate a feature selection ensemble to improve the classification performance. We hypothesize that methods that detect gait abnormalities more sensitively and measure early gait impairments would be better suited for fall detection.

## 2. Materials and Methods

### 2.1. Study Design

A sample of 1240 pwMS was recruited in the Multiple Sclerosis Center at the University Hospital Dresden from November 2020 until September 2021. Patients performed gait analyses according to the Dresden protocol for Multidimensional Walking Assessment (DMWA) [37] and answered the question of how often they have fallen in the last thirty days. The main inclusion criterion was the available response to the fall question. Patients without a clinical diagnosis of MS, pregnant patients and patients who were unable to walk, either with or without assistive devices, were excluded from the study. Written informed consent was obtained from the individuals for the publication of any potentially identifiable images or data included in this article. The studies involving human participants were reviewed and approved by the Ethics Committee at Technische Universität Dresden. Approval number: BO-EK-320062021.

### 2.2. Data Sets

In the present study, data were collected during the routine visit to our clinic by research nurses with the multidimensional gait analysis according to the DMWA protocol [37]. The multidimensional gait analysis comprises a systematic recording of human gait at different speeds under differentiated tasks with standardized test methods and additional balance testing while standing. 

Subjects were tested with the GAITRite (CIR-Systems Inc., Franklin, NJ, USA) system. The GAITRite is an instrumental gait analysis system for recording spatiotemporal gait parameters. It consists of a portable electronic walking mat equipped with pressure sensors, which measure foot pressure and graphically display and quantify the gait pattern by means of processing software [38]. Subjects had to walk twice, passing the 8-meter walkway at their self-selected walking speed. This measurement resulted in two data sets. The first data set included items of normal walking (*GR_N data set*), and the second one included items of walking while answering questions (*GR_D data set*).

Another system used for measuring balance and spatiotemporal gait parameters is the Mobility Lab (APDM Inc., Portland, OR, USA) [39,40]. Six individual sensor units were attached to the patient’s wrists, ankles, sternum and lower back for recording the patient while walking. Furthermore, patients performed normal (*ML_N data set*) and dual-task (*ML_D data set*) walking over an 8-meter walkway for recording walking quality. For testing postural sway, the Romberg stance was performed with open (*ML_S_EO data set*) and closed eyes (*ML_S_EC data set*) in a firm stance with the feet hip-width parallel and the hands supported on the hips. 

The widespread EDSS was used to assess the neurostatus of different functional systems (visual, brainstem, pyramidal, cerebellar, sensory, bowel bladder, cognition/fatigue, ambulation) [41]. The EDSS was assessed by a certified expert and depended on the rater’s subjective judgment [42]. Its subcategories of functional systems and total score formed the *EDSS data set*.

Two further assessments are the Timed 25-Foot Walk Test (T25-FW) and the 2-Minute Walk Test (2MWT). The most widely used and robust T25-FW was applied to determine walking speed over a distance of 7.62 m recorded with a stopwatch by a nurse [43]. Walking endurance was evaluated using the 2MWT by continuously recording the distance walked for 2 minutes using an odometer [44]. These two parameters, all functional scores as well as the EDSS score, were summarized in the *Basic data set*.

The patient’s subjective perception of increasing walking impairment was assessed with the 12-item Multiple Sclerosis Walking Scale (MSWS-12) (*MSWS-12 data set*) in which patients rate their limitations in walking ability due to MS during the past two weeks with a Likert-Scale (not at all, a little, moderately, quite a lot, extremely) [45]. The perception of already low impairment was measured with the 9-item Early Mobility Impairment Questionnaire (EMIQ) (*EMIQ data set*) by which patients describe their ability to walk in the past 30 days with a Likert-Scale (none of the time, some of the time, most of the time, all of the time) [17].

All 428 parameters for each data set are summarized in Table 1. In addition to these ten data sets, an 11th data set (*All data set*) was formed with all features.

### 2.3. Data Preprocessing

Data sets must be preprocessed before being applied to classification methods. Metric features were present in the data sets *GR_N*, *GR_D*, *ML_N*, *ML_D*, *ML_S_EO* and *ML_S_EC*. In case of missing values, features were imputed with median values. The MSWS-12 and the *EMIQ data set* are questionnaires and consist of ordinal features. These features were imputed with mode values in case of missing values. The *EDSS data set* consists of ordinal features. The EDSS score is determined using the functional scores (visual, brainstem, pyramidal, cerebellar, sensory, bowel/bladder, cognition/fatigue, ambulation). For missing values, we used knowledge-based imputation [41], or if not possible, we imputed with the mode value from patients with the same final EDSS score. The *Basic data set* consists of metric features and ordinal features of the *EDSS data set*. The metric features were also imputed with median values. All ordinal features have a natural order. Thus, we assumed these features as quasi-metric. Missing values of ordinal features were handled in the same way as the *EDSS data set*. All features were standardized before applying the classification models.

### 2.4. Feature Selection Ensemble

**Figure 1:**
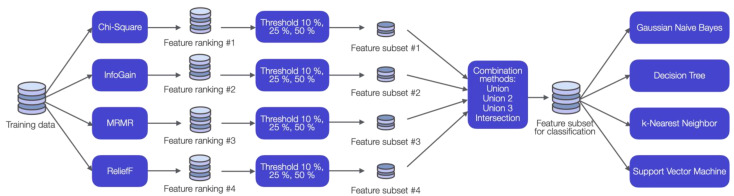
Workflow of the feature selection ensemble and the classification methods. Chi-square test (Chi-Square), Information gain (InfoGain), Minimum Redundancy Maximum Relevance (MRMR) and RelieF rank the features. Then, a threshold reduced this ranked list to a feature subset. A combination method combined these four feature subsets to a final feature subset for classification. Three different thresholds were investigated: 50%, 25%, 10%. Four different combination methods were investigated: Union, Union 2, Union 3, Intersection.

We wanted to investigate which of these eleven data sets (including the *All data set*) are suitable for fall detection using machine learning algorithms and explore the most important features of each data set. For this purpose, a feature selection ensemble (FS-Ensemble) was developed. Feature selection ensembles have successfully been applied in various publications [46,47,48,49]. The objective of our FS-Ensemble was to remove irrelevant and noisy features and thus increase the classification performance. The methodology is illustrated in Figure 1.

An ensemble benefits from different metrics used by each filter method to rate the features by relevance [50]. Therefore, we used four filter methods: Chi-square test (Chi-Square), information gain (InfoGain), Minimum Redundancy Maximum Relevance (MRMR) and RelieF. These methods are based on different metrics (statistical, information, mutual information, distance), which makes them suitable for an ensemble [46]. We limited the feature selection methods to filters because these methods are independent of a classification model in contrast to wrapper and embedded methods. As a result, these methods do not tend to overfit and will produce more general results than wrapper methods [32]. The filter methods Chi-Square, MRMR and RelieF were performed using MATLAB R2021b [51]. The filter method InfoGain was performed using WEKA 3.8 [52].

All four filter methods generated a ranked list of features. A threshold was applied to select a subset of features of each filter method (filter subsets). Then, a combination method combined the four filter subsets. In the literature, this approach showed a better performance than first applying a combination method and, subsequently, a threshold method [46,47].

Three thresholds and four combination methods were investigated according to a study by Seijo-Pardo et al. [46]. The thresholds were set to 50%, 25% or 10% and determined the number of features in each filter subset. The following combination methods were applied to merge the subsets:

- Union: A feature was selected for the final subset if this feature was present in at least one of the filter subsets.
- Union 2: A feature was selected in the final subset if this feature was in at least two of the filter subsets.
- Union 3: A feature was selected in the final subset if this feature was in at least three of the filter subsets.
- Intersection: A feature was selected in the final subset if this feature was in all of the filter subsets.

The final subset created in each process contains all input features used for the classification models. The combination methods were performed using Python version 3.8.8.

### 2.5. Classification Models

Four classification methods were applied: Gaussian Naive Bayes, Decision Tree, k-Nearest Neighbor and Support Vector Machine. The Classification and Regression Tree algorithm (CART algorithm) was used to build up the Decision Tree. The Radial Basis Function (RBF) kernel was used for training a Support Vector Machine. The parameters of the Decision Tree, the k-Nearest Neighbor and the Support Vector Machine were optimized using grid search. The ranges of the hyperparameters are shown in Table 2. A stratified 5-fold cross-validation with F1 score as evaluation score was implemented. F1 score is more suitable than the accuracy in the case of imbalanced data [53,54].

F1 score, recall, precision, specificity and Cohen´s kappa were calculated to evaluate and compare the different models based on the hyperparameter optimization using 5-fold cross-validation. The stratified 5-fold cross-validation was repeated 10 times to reduce bias when splitting the data into the folds.

A permutation test was performed on each model to verify how the results compare to random guessing [55]. The test performed 1000 permutations. Each permutation consists of data points with randomly chosen labels. This removed the dependency between features and labels. The *p*-value of this test was calculated to estimate whether the predictions were better than random guessing. Machine learning classification and evaluation were performed using Python version 3.8.8 and scikit-learn version 0.18.1 [56].

## 3. Results

### 3.1. Subject’s Characteristics

We had 1240 pwMS participate in the study; 911 were female (73.5%) and 329 males (26.5%) aged 17 to 80 (mean ± SD = 44.8 ± 12.2). Further, 172 (13.9%) pwMS stated having fallen. Thereby, age (*p* < 0.001, *t*-test) was a significant factor, as well as the type of therapy: no therapy, first-line therapy or second-line therapy (*p* < 0.001, Chi-square test). Participants reported a mean disease duration since diagnosis of 12.96 (SD = 57.68) years and a median EDSS of 3.0 (interquartile range IQR = 2.0). The median EDSS score was 2.0 (IQR = 2.5) for non-fallers and 5.0 (IQR = 3.0) for fallers. Table 3 summarizes the characteristics of the study participants.

Not all data sets were available for each patient due to mobility impairments, system errors or missing self-reports.

### 3.2. Feature Selection Ensemble and Classification

Four classification models were used to determine the most suitable method for fall detection. For a better overview, we focused on the best classification results for each data set. PROs (MSWS-12 and EMIQ) achieved the best F1 score (F1 = 0.54 ± 0.00). The *All data set* showed an almost equally good performance (F1 = 0.53 ± 0.00). In these three data sets, recall was higher than precision. Rater-based methods had a better F1 score (F1 = 0.48 ± 0.00 and F1 = 0.47 ± 0.01) than sensor-based methods. Among the sensor-based methods, the measurements with cognitive tasks showed worse F1 values (F1 = 0.43 ± 0.01 and F1 = 0.42 ± 0.00) than measurements during normal walking (F1 = 0.39 ± 0.00 and F1 = 0.38 ± 0.01). Balance tests showed the worst results (F1 = 0.30 ± 0.01 and F1 = 0.29 ± 0.02). Gaussian Naive Bayes was the best classification model for all data sets except for *ML_S_EO*. The k-Nearest Neighbor was the best model (F1 = 0.29 ± 0.02) for this method. However, the performance of this model was poorest in comparison to the other performances of the Gaussian Naive Bayes. *ML_S_EO* and *ML_S_EC* achieved the worst results with the Support Vector Machine (F1 = 0.20 ± 0.01 and F1 = 0.19 ± 0.02). All of the best-performing classification models of each data set generated a highly significant value (*p* ≤ 0.001) in the permutation test. 

**Figure 2:**
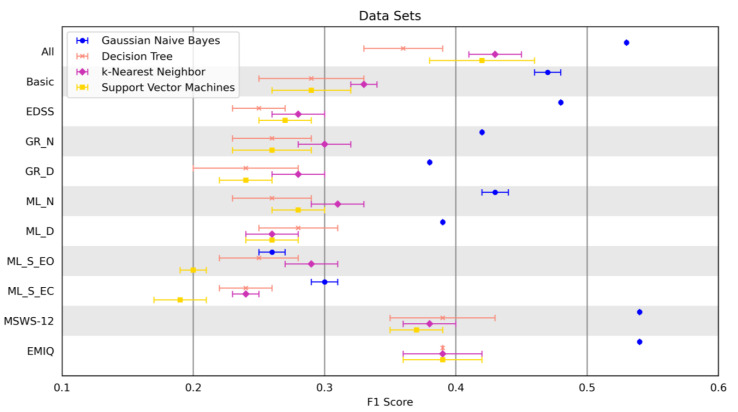
Best Performance (F1 score) of the four classification models on the detection of fall using different data sets. The error bars present the mean and standard deviation across 5-fold cross-validation repetition. *All data set* = All features of the following data sets; *Basic data set* = 25 Foot Walk Test + 2-minute walk test + EDSS; Expanded Disability Status Scale (EDSS); GAITRite System normal (*GR_N data set*) and dual-task (*GR_D data set*) walking; Mobility Lab Gait normal (*ML_N data set*) and dual-task (*ML_D data set*) walking; Mobility Lab Romberg stance with open (*ML_S_EO data set*) and closed eyes (*ML_S_EC data set*); Twelve Item Multiple Sclerosis Walking Scale (MSWS-12); Early Mobility Impairment Questionnaire (EMIQ).

The best results for the F1 score for each classification model are shown in Figure 2. The overall best performances for each data set are shown in Table 4. If two results showed the same F1 score for a data set, the result with fewer features was chosen. 

The optimized parameters for each classification model are shown in Appendix A (Table A1). Table A2, Table A3, Table A4, Table A5, Table A6, Table A7, Table A8, Table A9, Table A10, Table A11, and Table A12 in Appendix B show the classification results for all eleven data sets. The FS-Ensemble improved the classification performance in most cases or at least did not lead to any fallbacks. There was no threshold or combination method that always worked best, but often a restrictive selection improved the performance.

When using the data set with all 428 features (*All data set*), all models showed a better F1 score when reducing the number of features. The Gaussian Naive Bayes outperformed the other classification models in each experiment. Additionally, the method showed a consistently lower standard deviation. Decision Tree achieved the worst performances in most cases. The best performance for the Gaussian Naive Bayes classification model was achieved by reducing to 9 features (99% reduction), for the Decision Tree when limiting the number of features to 9 (99% reduction), for the Support Vector Machine when restricting to 25 (94% reduction) features and for the k-Nearest Neighbor when using 44 features (90% reduction).

**Figure 3:**
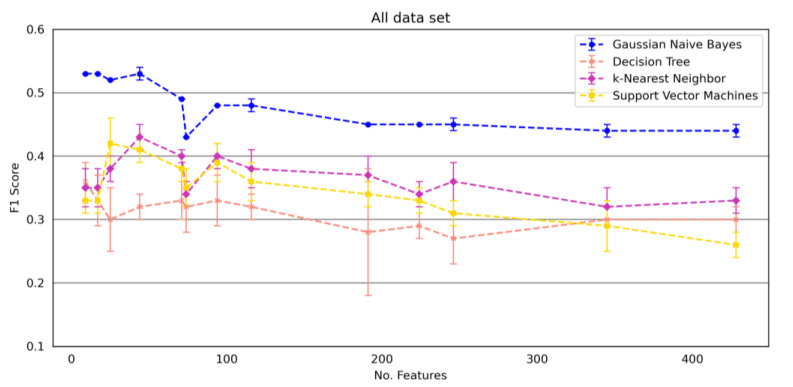
Classification performance (F1 score) of the four models on *All data set* depending on the number of features. The error bars present the mean and standard deviation across 5-fold cross-validation repetition. A dotted line maps the F1 score trend with increasing feature integer values. No assumption is made for the performance of feature subsets that lie between the data points. *All data set* = 25 Foot Walk Test + 2-minute walk test + Expanded Disability Status Scale + GAITRite System data sets + Mobility Lab Gait data sets + 12 item Multiple Sclerosis Walking Scale data set + Early Mobility Impairment Questionnaire data set.

Figure 3 shows the classification performances of the four classification models using all data (*All data set*) depending on the number of features.

After determining the best method for each data set, we considered the list of features selected in each method. Table 5 shows the final feature subsets that achieved the best performance according to Table 4.

Although only features from EMIQ and MSWS-12 were selected by the filter methods, the *All data set* achieved the highest recall (74.0%). Q1_EMIQ, Q4_EMIQ, Q8_EMIQ and Q12_MSWS-12 were selected in both final subsets: using all data and the individual data set. For detecting falls with the Gaussian Naive Bayes when using the *All data set*, the features addressing the patient´s judgment of walking, running and stair climbing ability, subjective effort as well as necessary concentration and walking fluency during walking, frequency of stumbling and indication of avoidance of social activities were used. For rater-based data sets (*Basic* and *EDSS*), all given features were used to achieve the best performance. GAITRite data sets concentrated on step count, velocity and step and stride length for the left and right leg, whereas under dual-task conditions, only step and stride length were used. The final feature subset of the Mobility Lab normal walking data set used for fall detection consisted of the percentage amount of the individual stride phases, the gait speed and the stride length of the right leg. Among the dual-task conditions, the number of used features was reduced to the stride length of the left leg, the percentage amount of the Terminal Double Support phase of the right leg and the degree of the toe-off angle of both legs. During balance testing with eyes open, the jerk trajectory and degree of the 95% Ellipse rotation were the best features for detecting falls. For balance testing with eyes closed, mostly all features were used. The final feature subset of the *MSWS-12 data set* included, just like the *All data set*, the concentration and estimation of the balance and needed walking aid at home. The final feature subset of the *EMIQ data set* included, just like the *All data set,* the concentration, frequency of stumbling and indication of avoidance of social activities. Furthermore, the indication of avoidance of sports activities was used.

## 4. Discussion

Our objective was to investigate which of the eleven data sets is suitable to detect falls and the most important features for this purpose. A new feature selection ensemble (FS-Ensemble) and four classification models were applied. The Questionnaires (MSWS-12 and EMIQ) achieved the best performance (F1 = 0.54 ± 0.00/0.01) and seemed most suitable for fall detection when using the Gaussian Naive Bayes. Thus, we are able to confirm our hypothesis that patients’ self-assessments can better reflect health status than other methods. PROs can provide more in-depth insights into a specific domain, are thought to be more sensitive to changes in health status, and questions may seem more relevant and, therefore, more acceptable to patients [13,57,58]. This statement is supported by the composition of the feature subset of the *All data set*, which consists exclusively of questionnaire parameters. A recall of 74% for fall detection can be attained if using a selection of features of MSWS-12 and EMIQ. 

Contrary to the assumption that balance parameters are more suitable in detecting falls, based upon a more sensitive recording of gait alterations compared to typical gait data [15,16], our results show that data sets with balance parameters (*ML_S_EO*, *ML_S_EC*) are less able to detect falls in pwMS (F1 = 0.30 ± 0.01 and F1 = 0.29 ± 0.02) instead of PROs and rater-based items with the used ML methods. Including further machine learning methods could still improve their F1 score. 

Rater-based methods showed the second-best performance after patient-based methods. One reason could be the examination of different bodily functions. Research on fall risk factors in inpatients identified twenty different intrinsic and extrinsic factors [59]. This suggests that including multiple factors, as collected with the EDSS, leads to a better result than using only sensor-based data. The performances were not improved when including the T25-FW and the 2MWT in addition to the EDSS score and the functional scores (*Basic data set*). Thus, these methods bring no added benefit but instead increase the effort of data collection. In this case, we do not recommend this combination of tests and EDSS scores when detecting falls with machine learning.

Sensor-based systems such as the GAITRite system or the Mobility Lab show the best F1 scores with the Gaussian Naive Bayes but seem to be unsuitable for fall detection compared to the questionnaires and rater-based methods. The systems are only occasionally used in routine clinical practice, and clinicians only consider a few parameters in the assessment. An automatic analysis of the results of these sensor systems would be a benefit for the users. First, this could save personnel capacities, and second, an evaluation of all available parameters would be possible. Therefore, in our opinion, further research with the sensor-based system used is promising. Additionally, extending the database can improve performance by collecting more sensor-based data from fallen patients. 

The Gaussian Naive Bayes outperformed the other classification methods and is suitable for handling these different data sets (Figure 2). Thus, we recommend using the Gaussian Naive Bayes for detecting falls in pwMS. The fewer features included in the calculations, the better the detection of falls (Figure 3).

Knowing that perceived fragility in the wake of fear of falling can lead to falls, we recommend including this factor in future studies. Fear of falling can reduce self-confidence, worsen physical health, including mobility, due to social withdrawal and physical inactivity, and subsequently lead to falls [8]. Therefore, some studies defined fear of falling as a risk factor for fall detection [60,61]. Thus, detecting fear of falling and appropriate treatment can reduce the risk of falling [8].

Our study is not without limitations. Self-reported questionnaires, as well as the question “How often have you fallen in the last thirty days?", always collect retrospective information, so a recall bias could impact the reporting of falls. A basic human need is independence. As reporting of falls indicates a lack of independence, MS patients may under-report their number of falls [10,62]. Bias could be reduced by recording falls more accurately via a diary, in which patients note falls daily. Furthermore, the use of longitudinal data would be interesting to predict falls.

Missing values are a major problem of studies, as they affect the significance of results. A natural selection of patients who were unable to complete gait tests due to the severity of their disease limits the validity of our results. Cognitive and balance tasks are more challenging since patients with increased cognitive impairment need more attention while walking in order to compensate for further disorders [14]. This may result in missing data in more severely impaired individuals and could explain the poor performance of the sensor-based data sets in our study (Figure 2) compared to other studies [14,15,16,17].

The data show significant differences between patients who fall and those who do not fall in relation to their age and how their MS disease is treated. To achieve better comparability of the data, a propensity score matching procedure would be beneficial. Due to the high number of patents, we included in our study (*N* = 1240), we have dispensed with this procedure.

Furthermore, the answer options for the questionnaires were coded from one to four or five. The questionnaire items own a natural order (ordinal features). Therefore, the items were assumed as quasimetric features in the preprocessing step to process all features equally and to use the same classification models. Thus, the results must be considered with caution. The numeric distances between the coded items could be unequal. Therefore, the results do not have to correspond to reality in this case, but this assumption is a common preprocessing step in statistics and machine learning [63,64]. 

Test sets were not used in our study due to the small data size. Therefore, the results could have a positive bias. We addressed this problem by only using filter methods in contrast to other studies [46,48,65]. Filter methods are more general than other methods and do not tend to overfit [35,66]. In addition, a stratified 5-fold cross-validation was used for the grid search and performance evaluation. This validation method was often used for small data sizes and to make the results more generally valid [67]. However, the split of the folds in the train and validation set is a significant issue for calculating and interpreting the validation score since the score could be obtained by chance [68]. Thus, the cross-validation was repeated 10 times for performance evaluation and preceded by a permutation test. These methods are sufficient to overcome the missing test sets.

The data sets in this study were imbalanced due to the nature of fall detection. The used machine learning methods are not suitable for handling these imbalanced data sets [69]. This problem can be reduced with feature selection to delete redundant and irrelevant features. Thus, the methods could achieve better performance. Our FS-Ensemble consisted of four filter methods. These filter methods are suitable to ensure diversity for the ensemble [36,55], but another ensemble configuration could increase the diversity and, thus, improve the classification performance. Seijo-Pardo et al. used an ensemble that consists of four filter methods and two embedded methods [46]. They summarized that the ensemble works better than the individual feature selection methods. Moghimi et al. investigated filter, wrapper and embedded methods as an ensemble [70]. A combination of each one of these selection methods achieved the best classification performance. 

The FS-Ensemble failed when the data set already consisted of very few features (e.g., *Basic*, *EDSS*, *EMIQ* and *MSWS12*). The nature of our feature selection method allows for the selection of few or even only one feature. This feature size would be insufficient for some of the machine learning algorithms in this study. Most machine learning methods are suitable for detecting patterns by considering feature dependencies. This benefit disappears when using a few or just one feature. Furthermore, the applied permutation test failed in some of these cases (*p* > 0.001). This means that the classification results occurred randomly and confirms that few or one features in a machine learning model are insufficient for providing valid results. Since some classification models can handle a single feature within the data set, the same method was applied throughout all machine learning algorithms for completeness to detect the most suitable one. For this purpose, the FS-Ensemble was an appropriate method. But we recommend using a higher threshold and the union combination method on data sets with few features.

## 5. Conclusions

We investigated eleven data sets derived from widely used gait assessment systems and PRO questionaries for mobility assessment to evaluate their predictive power for the detection of falling in pwMS. We can confirm that patients’ self-assessments can better reflect health status than other methods. Therefore, easy to use questionnaires (MSWS-12 and EMIQ) can provide a highly cost-effective tool for day-to-day clinical praxis when assisted by a sufficient trained machine learning algorithm. These kinds of algorithms could be implemented in AI-based medical software with very little effort compared to sensor-based assessment systems. Nevertheless, sensor data allows additional in-depth insights into the specific impairment as the root cause of the fall risk and possible starting point for a successful treatment. 

The FS-Ensemble is appropriate to improve the classification performance using data sets with more than 30 features. Further research is needed to investigate whether classification performance can be improved and generalized with more data from fallen patients. Additionally, the inclusion of more risk factors of falling, such as fear of falling, must be investigated for preventing falls. 

## Figures and Tables

**Table 1:** Mobility and clinical parameters recorded by the physician or nurse, self-reported gait parameters by patients as well as gait parameters recorded by gait analysis systems.

Data Set	No.	Features
*Basic data set*	11	Walking speed (T25-FW), walking endurance (2MWT), EDSS subcategories of functional systems (visual, brainstem, pyramidal, cerebellar, sensory, bowel bladder, cognition/fatigue, ambulation) and total score
*EDSS data set*	9	EDSS subcategories of functional systems (visual, brainstem, pyramidal, cerebellar, sensory, bowel bladder, cognition/fatigue, ambulation) and total score
GAITRite System (*GR_N* and *GR_D data set*)	82	Ambulation Time, Cadence, Cycle Time, Walking Distance, Foot Length, Foot Width, Functional Ambulation Profile, Leg Length, Step Count, Step Length, Step Time, Stride Length, Stride Time, Stride Velocity, walking velocity, various parameters of the individual gait phases [38]
Mobility Lab Gait (*ML_N* and *ML_D data set*)	84	Duration, Cadence, Circumduction, Foot Strike Angle, Gait Cycle Duration, Gait Speed, Lateral Step Variability, Stance duration, Step Duration, Stride Length, Toe Off Angle, Coronal Range of Motion, Sagittal Range of Motion, Transverse Range of Motion, Turns Angle, Turns Duration, Steps in Turn, Turn Velocity, Arm Range of Motion, Arm Swing Velocity [39]
Mobility Lab Postural Sway (*ML_S_EO* and *ML_S_EC data set*)	32	Ellipse Axis, Ellipse Rotation, Range of sway in the sagittal and coronal plane, anterior-posterior and mediolateral trunk fluctuation range, Sway Velocity [39]
*MSWS-12 data set*	12	Self report: Limitation of walking ability, Limitation of balance, Limitation of walking distance, Effort for walking, Need of walking aids, Limitation of walking velocity [45]
*EMIQ data set* In the past 30 days, how often have you…	9	Self report: Walking ability without/during cognitive tasks, Need of walking aid, Limitation of walking velocity, Limitation of household, social and physical activity [17]
*All data set*	428	All features of the *Basic data set*, the GAITRite System data sets, the Mobility Lab Gait data sets, the *MSWS-12 data set* and the *EMIQ data set*

25 Foot Walk Test (T25-FW), 2-minute walk test (2MWT), Expanded Disability Status Scale (EDSS); Twelve Item Multiple Sclerosis Walking Scale (MSWS-12); Early Mobility Impairment Questionnaire (EMIQ). GAITRite System normal (*GR_N data set*) and dual-task (*GR_D data set*) walking; Mobility Lab Gait normal (*ML_N data set*) and dual-task (*ML_D data set*) walking; Mobility Lab Romberg stance with open (*ML_S_EO data set*) and closed eyes (*ML_S_EC data set*).

**Table 2:** Range of hyperparameters optimized with grid search.

Method	Hyperparameter	Min	Max	Step Size	Scale
Decision Tree	Criterion: ‘gini’ or ‘entropy’	-	-	-	-
	Maximum depth	2	10	1	linear
	Minimum samples at a leaf node	5	30	1	linear
k-Nearest Neighbor	Weights: ‘uniform’ or ‘distance’	-	-	-	-
	Distance metric: ‘euclidean’ or ‘manhattan’	-	-	-	-
	Numbers of neighbors	2	10	1	linear
Support Vector Machine	Regularization C	0.1	100	10	logarithmic
	Kernel coefficient gamma	10^−5^	1	10	logarithmic

**Table 3:** Demographic and clinical characteristics of the patients (*N* = 1240). First-line therapies subsume the disease-modifying drugs azathioprine, Glatirameracetate, Dimethylfumarat, Interferons and Teriflunomide. Second-line therapies subsume the high-efficacy MS treatments Alemtuzumab, Cladribine, S1P Modulators, Natalizumab, Anti-CD20 treatments.

	Variable	Participants, No. (%)
Gender	Female	911 (73.5)
	Male	329 (26.5)
MS subtype	Relapsing-remitting	1091 (88.0)
	Primary progressive	83 (6.7)
	Secondary progressive	65 (5.2)
	MS subtype still unclear	1 (0.1)
Immunomodulatory Therapies	First-line therapies	357 (28.8)
	Second-line therapies	629 (50.7)
	No therapy	254 (20.5)

Multiple Sclerosis (MS).

**Table 4:** Performances of the best classification model per data set on detecting falls. The values are presented as mean ± standard deviation across 5-fold cross-validation repetition. *p*-value via permutation test.

	Thresh-Old	Combination Method	No. Features	F1	Recall (%)	Precision (%)	Specificity (%)	Kappa	*p*
***All data set *** **(*n* = 929)**									
**GNB**	10%	Intersection	9	0.53 ± 0.00	74.0 ± 0.0	41.0 ± 0.3	83.1 ± 0.2	0.43 ± 0.00	0.001
***Basic data set *** **(*n* = 1237)**									
**GNB**	100%	-	11	0.47 ± 0.01	57.6 ± 0.9	39.4 ± 0.4	85.7 ± 0.2	0.36 ± 0.01	0.001
***EDSS data set *** **(*n* = 1237)**									
**GNB**	100%	-	9	0.48 ± 0.00	64.6 ± 0.3	38.2 ± 0.2	83.1 ± 0.1	0.37 ± 0.00	0.001
***GR_N data set *** **(*n* = 1212)**									
**GNB**	50%	Intersection	7	0.42 ± 0.00	45.4 ± 0.6	38.4 ± 0.3	88.3 ± 0.1	0.31 ± 0.00	0.001
***GR_D data set *** **(*n* = 1213)**									
**GNB**	25%	Intersection	4	0.38 ± 0.01	41.0 ± 0.8	35.7 ± 0.4	88.5 ± 0.1	0.28 ± 0.01	0.001
***ML_N data set *** **(*n* = 1006)**									
**GNB**	10%	Union 2	8	0.43 ± 0.01	42.9 ± 0.6	43.1 ± 0.7	90.8 ± 0.2	0.34 ± 0.01	0.001
***ML_D data set *** **(*n* = 971)**									
**GNB**	25%	Intersection	4	0.39 ± 0.00	40.7 ± 0.6	37.4 ± 0.5	89.2 ± 0.1	0.29 ± 0.01	0.001
***ML_S_EO data set *** **(*n* = 1201)**									
**kNN**	25%	Union 3	2	0.29 ± 0.02	24.8 ± 1.5	35.0 ± 1.6	92.7 ± 0.3	0.20 ± 0.02	0.001
***ML_S_EC data set *** **(*n* = 1190)**									
**GNB**	25%	Union	22	0.30 ± 0.01	23.4 ± 0.9	40.8 ± 1.3	94.6 ± 0.2	0.22 ± 0.01	0.001
***MSWS-12 data set *** **(*n* = 1227)**									
**GNB**	25%	Union 2	3	0.54 ± 0.00	65.9 ± 0.0	45.4 ± 0.1	87.3 ± 0.0	0.45 ± 0.00	0.001
***EMIQ data set *** **(*n* = 1239)**									
**GNB**	25%	Union	4	0.54 ± 0.01	61.0 ± 1.4	48.0 ± 0.5	89.4 ± 0.2	0.45 ± 0.01	0.001

Gaussian Naive Bayes (GNB); k-Nearest Neighbor (kNN). *All data set* = All features of the following data sets; *Basic data set* = 25 Foot Walk Test + 2-minute walk test + EDSS; Expanded Disability Status Scale (EDSS); GAITRite System normal (*GR_N data set*) and dual-task (*GR_D data set*) walking; Mobility Lab Gait normal (*ML_N data set*) and dual-task (*ML_D data set*) walking; Mobility Lab Romberg stance with open (*ML_S_EO data set*) and closed eyes (*ML_S_EC data set*); Twelve Item Multiple Sclerosis Walking Scale (MSWS-12); Early Mobility Impairment Questionnaire (EMIQ).

**Table 5:** The final feature subsets achieve the best performance according to Table 4. The features, highlighted in bold, were chosen as the most relevant features in both the *All data set* and the individual data sets. The unit of each gait parameter is shown in parentheses. The parameters have been recorded and merged for the left and right sides (L/R). The parameters are presented as mean [mean] or standard deviation [std].

	Features	Used No./Total No.
***All data set***		
**GNB**	**Q1_EMIQ, Q4_EMIQ, Q8_EMIQ**, Q1_MSWS-12, Q2_MSWS-12, Q3_MSWS-12, Q7_MSWS-12, Q11_MSWS-12, **Q12_MSWS-12**	9/428
***Basic data set***		
**GNB**	T25-FW, 2MWT, EDSS_Ambulation, EDSS_Bowel_Bladder, EDSS_Brainstem, EDSS_Cerebellar, EDSS_cognition/fatigue, EDSS_Pyramidal, EDSS_Score, EDSS_Sensory, EDSS_Visual	11/11
***EDSS data set***		
**GNB**	EDSS_Ambulation, EDSS_Bowel_Bladder, EDSS_Brainstem, EDSS_Cerebellar, EDSS_cognition/fatigue, EDSS_Pyramidal, EDSS_Score, EDSS_Sensory, EDSS_Visual	9/9
***GR_N data set***		
**GNB**	Step Count, Step Length (cm) L, Step Length (cm) R, Stride Length (cm) L, Stride Length (cm) R, Stride Velocity Right, Velocity	7/82
***GR_D data set***		
**GNB**	Step Extremity (ratio) L, Step Length (cm) L, Step Length (cm) R, Stride Length (cm) R	4/82
***ML_N data set***		
**GNB**	Lower Limb – Double Support L (%GCT) [mean], Lower Limb – Gait Speed L (m/s) [mean], Lower Limb – Gait Speed R (m/s) [mean], Lower Limb – Single Limb Support L (%GCT) [mean], Lower Limb – Stance R (%GCT) [mean], Lower Limb – Swing R (%GCT) [mean], Lower Limb – Terminal Double Support R (%GCT) [std], Stride Length R (m) [mean]	8/84
***ML_D data set***		
**GNB**	Lower Limb – Stride Length R (m) [mean], Lower Limb – Terminal Double Support R (%GCT) [mean], Lower Limb – Toe Off Angle L (degrees) [mean], Lower Limb – Toe Off Angle R (degrees) [mean]	4/84
***ML_S_EO data set***		
**kNN**	Acc – Jerk (Sagittal) (m^2^/s^5^); Angles – 95% Ellipse Axis 1 Radius (degrees)	2/32
***ML_S_EC data set***		
**GNB**	Acc – 95% Ellipse Axis 1 Radius (m/s^2^), Acc – 95% Ellipse Rotation (radians), Acc – Centroidal Frequency (Hz), Acc – Centroidal Frequency (Coronal) (Hz), Acc – Centroidal Frequency (Sagittal) (Hz), Acc – Frequency Dispersi on (AD), Acc – Frequency Dispersion (Coronal) (AD), Acc – Frequency Dispersion (Sagittal) (AD), Acc – Jerk (m^2^/s^5^), Acc – Jerk (Coronal) (m^2^/s^5^), Acc – Jerk (Sagittal) (m^2^/s^5^), Acc – Mean Velocity (Coronal) (m/s), Acc – Path Length (m/s^2^), Acc – Path Length (Coronal) (m/s^2^), Acc – Path Length (Sagittal) (m/s^2^), Acc – Range (m/s^2^), Acc – Range (Coronal) (m/s^2^), Acc – RMS Sway (Coronal) (m/s^2^), Acc – RMS Sway (Sagittal) (m/s^2^), Angles – 95% Ellipse Axis 1 Radius (degrees), Angles – 95% Ellipse Rotation (radians), Angles – RMS Sway (Coronal) (degrees)	22/32
***MSWS-12 data set***		
**GNB**	Q5_MSWS-12, Q8_MSWS-12, **Q12_MSWS-12**	3/12
***EMIQ data set***		
**GNB**	**Q1_EMIQ, Q4_EMIQ, Q8_EMIQ**, Q9_EMIQ	4/9

Gait cycle time (GCT); Acceleration (Acc); root mean square (RMS); Q() = number of questions; *All data set* = All features of the following data sets; *Basic data set* = 25 Foot Walk Test (T25-FW) + 2-minute walk test (2MWT) + EDSS; Expanded Disability Status Scale (EDSS); GAITRite System normal (*GR_N data set*) and dual-task (*GR_D data set*) walking; Mobility Lab Gait normal (*ML_N data set*) and dual-task (*ML_D data set*) walking; Mobility Lab Romberg stance with open (*ML_S_EO data set*) and closed eyes (*ML_S_EC data set*); Twelve Item Multiple Sclerosis Walking Scale (MSWS-12); Early Mobility Impairment Questionnaire (EMIQ).

## Data Availability

The data presented in this study are available on reasonable request from the corresponding author.

## Appendix A

**Table A1:** Results of the hyperparameter optimization for each data set; Decision Tree (DT); k-Nearest Neighbor (kNN); Support Vector Machine (SVM); Euclidean (Eucl.); Manhattan (Manh.). Expanded Disability Status Scale (EDSS); *Basic data set* = 25 Foot Walk Test + 2-minute walk test + EDSS; *All data set* = All features of the *Basic data set*, the GAITRite System data sets, the Mobility Lab Gait data sets, the Twelve Item Multiple Sclerosis Walking Scale *data set* and the Early Mobility Impairment Questionnaire *data set*.

	Parameters	Baseline	Threshold 10%				Threshold 25%				Threshold 50%			
			Union	Union2	Union3	Inter-section	Union	Union2	Union3	Inter-section	Union	Union2	Union3	Inter-section
***All data set***														
**DT**	Criterion:	Entropy	Entropy	Gini	Entropy	Gini	Gini	Entropy	Gini	Entropy	Gini	Entropy	Gini	Entropy
	Maximum depth:	8	8	4	6	4	6	10	7	7	8	3	2	9
	Minimum:	10	15	6	11	29	16	10	6	9	5	22	15	13
**kNN**	Weights:	Distance	Distance	Distance	Distance	Uniform	Distance	Distance	Distance	Distance	Distance	Uniform	Uniform	Uniform
	Distance metric:	Eucl.	Eucl.	Eucl.	Eucl.	Manh.	Eucl.	Eucl.	Eucl.	Manh.	Eucl.	Eucl.	Eucl.	Eucl.
	No. neighbors:	2	10	6	10	3	2	4	4	4	4	3	3	3
**SVM**	Regularization C:	10	10	10	100	100	100	100	100	10	100	100	100	100
	Kernel coefficient γ:	0.001	0.01	0.01	0.01	0.1	0.001	0.001	0.01	0.1	0.001	0.001	0.001	0.01
***Basic data set***														
**DT**	Criterion:	Gini	Entropy	Gini	Gini	-	Gini	Entropy	Gini	-	Gini	Gini	Entropy	Gini
	Maximum depth:	7	7	5	5	-	4	6	5	-	7	10	10	2
	Minimum:	20	14	5	5	-	21	19	5	-	20	9	5	5
**kNN**	Weights:	Distance	Uniform	Uniform	Uniform	-	Distance	Uniform	Uniform	-	Distance	Distance	Distance	Uniform
	Distance metric:	Manh.	Eucl.	Eucl.	Eucl.	-	Manh.	Eucl.	Eucl.	-	Manh.	Eucl.	Manh.	Eucl.
	No. neighbors:	2	3	3	3	-	3	5	3	-	2	2	2	9
**SVM**	Regularization C:	100	100	1	1	-	100	100	1	-	100	100	100	0.1
	Kernel coefficient γ:	0.1	1	1	1	-	0.1	1	1	-	0.1	0.1	1	0.0001
***EDSS data set***														
**DT**	Criterion:	Gini	Entropy	Gini	Gini	-	Gini	Entropy	Gini	-	Gini	Gini	Entropy	Gini
	Maximum depth:	6	7	5	5	-	8	6	5	-	6	6	8	2
	Minimum:	21	14	5	5	-	12	19	5	-	21	21	7	5
**kNN**	Weights:	Distance	Uniform	Uniform	Uniform		Uniform	Uniform	Uniform	-	Distance	Distance	Distance	Uniform
	Distance metric:	Eucl.	Eucl.	Eucl.	Eucl.	-	Eucl.	Manh.	Eucl.	-	Eucl.	Eucl.	Eucl.	Eucl.
	No. neighbors:	2	3	3	3	-	5	3	3	-	2	3	3	9
**SVM**	Regularization C:	100	100	1	1	-	100	100	1	-	100	10	100	0.1
	Kernel coefficient γ:	0.1	1	1	1	-	1	1	1	-	0.1	1	1	0.0001
***GR_N data set***														
**DT**	Criterion:	Entropy	Gini	Entropy	Entropy	-	Entropy	Gini	Gini	Entropy	Entropy	Gini	Entropy	Gini
	Maximum depth:	10	7	10	10	-	9	9	5	8	10	10	9	5
	Minimum:	11	9	7	9	-	11	17	30	11	12	14	15	24
**kNN**	Weights:	Distance	Uniform	Distance	Distance	-	Distance	Distance	Distance	Distance	Distance	Distance	Distance	Distance
	Distance metric:	Manh.	Manh.	Eucl.	Manh.	-	Eucl.	Manh.	Manh.	Manh.	Manh.	Manh.	Manh.	Manh.
	No. neighbors:	2	3	2	3	-	2	2	2	3	2	2	2	2
**SVM**	Regularization C:	10	100	100	100	-	100	100	100	100	100	100	100	100
	Kernel coefficient γ:	0.01	0.01	1	1	-	0.01	0.01	1	1	0.01	0.01	0.1	1
***GR_D data set***														
**DT**	Criterion:	Entropy	Gini	Entropy	Entropy	-	Entropy	Entropy	Entropy	Gini	Entropy	Entropy	Gini	Gini
	Maximum depth:	8	9	10	10	-	6	9	10	10	10	10	8	9
	Minimum:	17	9	9	5	-	22	5	9	5	21	6	10	8
**kNN**	Weights:	Uniform	Distance	Uniform	Distance	-	Distance	Distance	Uniform	uniform	Distance	Distance	Distance	Uniform
	Distance metric:	Manh.	Manh.	Eucl.	Eucl.	-	Eucl.	Manh.	Manh.	Eucl.	Manh.	Eucl.	Eucl.	Manh.
	No. neighbors:	5	2	2	2	-	5	2	3	3	2	2	2	3
**SVM**	Regularization C:	10	10	100	100	-	100	10	100	100	100	100	100	100
	Kernel coefficient γ:	0.01	0.1	0.1	1	-	0.01	0.1	1	1	0.01	0.01	0.01	0.1
***ML_N data set***														
**DT**	Criterion:	Entropy	Gini	Gini	Gini	-	Gini	Gini	Gini	Entropy	Gini	Gini	Gini	Entropy
	Maximum depth:	6	2	4	9	-	2	2	5	7	5	2	9	3
	Minimum:	8	11	18	8	-	14	30	13	14	11	11	7	30
**kNN**	Weights:	Distance	Distance	Distance	Uniform	-	Distance	Distance	Uniform	Distance	Distance	Distance	Distance	Distance
	Distance metric:	Manh.	Eucl.	Eucl.	Eucl.	-	Manh.	Eucl.	Eucl.	Manh.	Eucl.	Eucl.	Eucl.	Eucl.
	No. neighbors:	2	2	2	3	-	2	2	3	2	2	2	2	2
**SVM**	Regularization C:	10	100	100	1	-	100	100	100	100	100	100	100	100
	Kernel coefficient γ:	0.01	0.01	0.1	1	-	0.01	0.1	0.1	1	0.01	0.01	0.01	0.1
***ML_D data set***														
**DT**	Criterion:	Entropy	Gini	Gini	Entropy	Gini	Gini	Entropy	Gini	Gini	Entropy	Entropy	Gini	Entropy
	Maximum depth:	6	6	6	10	9	10	6	6	6	5	5	6	7
	Minimum:	15	5	17	7	10	5	14	16	9	15	15	15	11
**kNN**	Weights:	Distance	Distance	Distance	Distance	Distance	Distance	Distance	Distance	Distance	Distance	Distance	Distance	Uniform
	Distance metric:	Eucl.	Manh.	Manh.	Manh.	Eucl.	Eucl.	Manh.	Manh.	Eucl.	Eucl.	Eucl.	Manh.	Manh.
	No. neighbors:	2	4	2	2	4	2	2	2	2	2	2	2	3
**SVM**	Regularization C:	100	10	100	100	10	100	10	100	100	100	100	100	100
	Kernel coefficient γ:	0.01	0.1	0.0001	0.0001	1	0.01	0.1	0.1	1	0.01	0.01	0.01	0.1
***ML_S_EO data set***														
**DT**	Criterion:	Gini	Entropy	Gini	Entropy	-	Gini	Gini	Entropy	-	Entropy	Entropy	Gini	-
	Maximum depth:	10	8	10	6	-	6	9	9	-	7	10	9	-
	Minimum:	16	6	7	7	-	17	14	7	-	14	10	7	-
**kNN**	Weights:	Distance	Distance	Distance	Uniform	-	Distance	Distance	Distance	-	Distance	Distance	Distance	-
	Distance metric:	Manh.	Eucl.	Eucl.	Eucl.	-	Manh.	Eucl.	Eucl.	-	Manh.	Eucl.	Manh.	-
	No. neighbors:	2	3	2	9	-	6	2	4	-	4	2	2	-
**SVM**	Regularization C:	100	10	100	100	-	100	100	100	-	10	100	100	-
	Kernel coefficient γ:	0.1	1	1	1	-	0.1	1	1	-	0.1	0.1	1	-
***ML_S_EC data set***														
**DT**	Criterion:	Entropy	Gini	Gini	-	-	Entropy	Gini	Gini	-	Gini	Gini	Gini	Gini
	Maximum depth:	9	5	5	-	-	7	7	3	-	5	7	7	9
	Minimum:	7	7	21	-	-	7	6	21	-	20	10	15	5
**kNN**	Weights:	Uniform	Distance	Distance	-	-	Distance	Distance	Uniform	-	Distance	Distance	Uniform	Distance
	Distance metric:	Eucl.	Eucl.	Manh.	-	-	Eucl.	Manh.	Eucl.	-	Eucl.	Eucl.	Manh.	Manh.
	No. neighbors:	3	2	4	-	-	2	2	3	-	3	2	3	2
**SVM**	Regularization C:	100	100	10	-	-	100	100	10	-	100	100	100	10
	Kernel coefficient γ:	0.1	0.1	1	-	-	0.1	1	1	-	0.1	0.1	1	1
***MSWS-12 data set***														
**DT**	Criterion:	Entropy	Entropy	Gini	Gini	-	Entropy	Entropy	Gini	Gini	Entropy	Gini	Gini	Gini
	Maximum depth:	7	5	2	2	-	4	6	2	2	4	4	3	2
	Minimum:	24	14	5	5	-	7	5	5	5	7	5	5	5
**kNN**	Weights:	Uniform	Uniform	Uniform	Uniform	-	Uniform	Uniform	Uniform	Uniform	Uniform	Distance	Distance	Uniform
	Distance metric:	Manh.	Eucl.	Eucl.	Eucl.	-	Manh.	Manh.	Eucl.	Eucl.	Eucl.	Eucl.	Manh.	Eucl.
	No. neighbors:	3	3	3	3	-	3	9	3	3	7	10	3	3
**SVM**	Regularization C:	100	1	0.1	0.1	-	100	100	0.1	0.1	100	100	10	0.1
	Kernel coefficient γ:	0.1	1	0.0001	0.0001	-	0.1	0.1	0.0001	0.0001	0.1	0.1	1	0.0001
***EMIQ data set***														
**DT**	Criterion:	Gini	Gini	Gini	Gini	-	Entropy	Gini	Gini	-	gini	gini	Gini	gini
	Maximum depth:	2	4	3	3	-	7	6	3	-	2	2	2	3
	Minimum:	7	27	5	5	-	13	5	5	-	7	15	15	5
**kNN**	Weights:	Distance	Uniform	Uniform	Uniform	-	Uniform	Uniform	Uniform	-	Uniform	Uniform	Uniform	Distance
	Distance metric:	Manh.	Eucl.	Eucl.	Eucl.	-	Eucl.	Eucl.	Eucl.	-	Manh.	Manh.	Manh.	Eucl.
	No. neighbors:	7	7	9	9	-	3	5	9	-	9	9	9	5
**SVM**	Regularization C:	10	100	1	1	-	10	10	1	-	10	10	10	10
	Kernel coefficient γ:	0.1	0.1	1	1	-	1	1	1	-	0.1	1	1	1

## Appendix B

**Table A2:** Performance of the four classification models on detection fall using all data; Gaussian Naive Bayes (GNB); Decision Tree (DT); k-Nearest Neighbor (kNN); Support Vector Machine (SVM); *p*-value via permutation test.

	Thresh-Old	Combination Method	No. Features	F1	Recall (%)	Precision (%)	Specificity (%)	Kappa	*p*
**GNB**	100%	-	428	0.44 ± 0.01	50.9 ± 1.0	38.1 ± 0.8	86.9 ± 0.3	0.33 ± 0.01	0.001
	50%	Union	345	0.44 ± 0.01	50.7 ± 0.8	38.4 ± 0.7	87.1 ± 0.3	0.33 ± 0.01	0.001
		Union 2	246	0.45 ± 0.01	51.8 ± 0.6	40.1 ± 0.7	87.7 ± 0.3	0.35 ± 0.01	0.001
		Union 3	191	0.45 ± 0.00	51.6 ± 0.8	39.9 ± 0.4	87.7 ± 0.2	0.35 ± 0.01	0.001
		Intersection	74	0.43 ± 0.00	53.6 ± 0.7	36.2 ± 0.4	85.1 ± 0.2	0.32 ± 0.01	0.001
	25%	Union	224	0.45 ± 0.00	54.4 ± 0.7	38.6 ± 0.3	86.3 ± 0.2	0.35 ± 0.00	0.001
		Union 2	116	0.48 ± 0.01	59.1 ± 0.6	40.1 ± 0.6	86.0 ± 0.3	0.38 ± 0.01	0.001
		Union 3	71	0.49 ± 0.00	63.6 ± 0.6	39.7 ± 0.2	84.7 ± 0.1	0.39 ± 0.00	0.001
		Intersection	17	0.53 ± 0.00	74.4 ± 0.4	41.2 ± 0.2	83.2 ± 0.1	0.43 ± 0.00	0.001
	10%	Union	94	0.48 ± 0.00	63.6 ± 0.6	38.9 ± 0.4	84.2 ± 0.2	0.38 ± 0.01	0.001
		Union 2	44	0.53 ± 0.01	72.9 ± 0.8	41.5 ± 0.4	83.7 ± 0.2	0.43 ± 0.01	0.001
		Union 3	25	0.52 ± 0.00	73.8 ± 0.5	40.4 ± 0.3	82.8 ± 0.2	0.42 ± 0.00	0.001
		Intersection	9	0.53 ± 0.00	74.0 ± 0.0	41.0 ± 0.3	83.1 ± 0.2	0.43 ± 0.00	0.001
**DT**	100%	-	428	0.30 ± 0.02	28.9 ± 2.0	30.6 ± 3.1	89.5 ± 1.4	0.19 ± 0.03	0.001
	50%	Union	345	0.30 ± 0.05	28.4 ± 5.1	31.7 ± 4.1	90.3 ± 1.0	0.20 ± 0.05	0.001
		Union 2	246	0.27 ± 0.04	20.9 ± 4.0	38.9 ± 1.4	94.8 ± 1.0	0.19 ± 0.03	0.001
		Union 3	191	0.28 ± 0.10	22.4 ± 10.7	40.8 ± 3.6	95.0 ± 2.2	0.21 ± 0.08	0.001
		Intersection	74	0.32 ± 0.04	27.2 ± 3.9	37.6 ± 3.7	92.8 ± 1.0	0.23 ± 0.04	0.001
	25%	Union	224	0.29 ± 0.02	25.2 ± 2.7	35.4 ± 2.9	92.7 ± 1.2	0.20 ± 0.02	0.001
		Union 2	116	0.32 ± 0.02	30.2 ± 2.7	34.5 ± 3.1	90.9 ± 1.2	0.22 ± 0.03	0.001
		Union 3	71	0.33 ± 0.03	29.1 ± 3.5	37.4 ± 2.9	92.2 ± 1.0	0.24 ± 0.03	0.001
		Intersection	17	0.33 ± 0.04	28.3 ± 4.1	41.3 ± 3.8	93.6 ± 1.1	0.25 ± 0.04	0.001
	10%	Union	94	0.33 ± 0.04	29.8 ± 5.1	38.5 ± 3.6	92.5 ± 0.9	0.25 ± 0.04	0.001
		Union 2	44	0.32 ± 0.02	26.1 ± 2.4	43.1 ± 2.8	94.5 ± 0.6	0.25 ± 0.02	0.001
		Union 3	25	0.30 ± 0.05	24.9 ± 5.7	39.1 ± 3.2	93.9 ± 1.3	0.22 ± 0.04	0.001
		Intersection	9	0.36 ± 0.03	27.7 ± 2.7	50.5 ± 3.1	95.7 ± 0.3	0.29 ± 0.03	0.001
**kNN**	100%	-	428	0.33 ± 0.02	32.3 ± 2.2	34.1 ± 1.3	90.1 ± 0.5	0.23 ± 0.02	0.001
	50%	Union	345	0.32 ± 0.03	25.5 ± 2.6	41.5 ± 3.0	94.3 ± 0.6	0.24 ± 0.03	0.001
		Union 2	246	0.36 ± 0.03	29.8 ± 3.2	45.2 ± 4.6	94.2 ± 0.8	0.28 ± 0.04	0.001
		Union 3	191	0.37 ± 0.03	31.7 ± 3.3	43.6 ± 3.7	93.5 ± 0.8	0.28 ± 0.04	0.001
		Intersection	74	0.34 ± 0.02	29.1 ± 2.3	41.8 ± 2.1	93.6 ± 0.6	0.26 ± 0.02	0.001
	25%	Union	224	0.34 ± 0.02	33.1 ± 2.3	34.9 ± 1.5	90.2 ± 0.6	0.24 ± 0.02	0.001
		Union 2	116	0.38 ± 0.03	33.5 ± 2.7	43.5 ± 3.4	93.1 ± 0.7	0.29 ± 0.03	0.001
		Union 3	71	0.40 ± 0.01	37.5 ± 1.2	43.6 ± 1.7	92.3 ± 0.5	0.32 ± 0.01	0.001
		Intersection	17	0.35 ± 0.03	32.0 ± 3.1	38.7 ± 2.2	92.0 ± 0.5	0.26 ± 0.03	0.001
	10%	Union	94	0.40 ± 0.02	31.7 ± 2.2	52.5 ± 3.1	95.4 ± 0.4	0.33 ± 0.03	0.001
		Union 2	44	0.43 ± 0.02	39.4 ± 2.8	48.1 ± 1.8	93.3 ± 0.3	0.35 ± 0.03	0.001
		Union 3	25	0.38 ± 0.02	32.1 ± 2.3	47.8 ± 2.5	94.4 ± 0.4	0.31 ± 0.02	0.001
		Intersection	9	0.35 ± 0.03	32.1 ± 2.5	38.9 ± 2.7	92.0 ± 0.4	0.26 ± 0.03	0.001
**SVM**	100%	-	428	0.26 ± 0.02	20.4 ± 1.9	35.5 ± 2.7	94.1 ± 0.6	0.18 ± 0.02	0.001
	50%	Union	345	0.29 ± 0.04	27.1 ± 4.8	32.1 ± 4.2	91.0 ± 0.9	0.19 ± 0.05	0.001
		Union 2	246	0.31 ± 0.02	28.0 ± 2.4	36.0 ± 2.6	92.1 ± 0.6	0.22 ± 0.03	0.001
		Union 3	191	0.34 ± 0.02	28.9 ± 2.2	41.0 ± 2.8	93.4 ± 0.5	0.25 ± 0.03	0.001
		Intersection	74	0.35 ± 0.03	32.3 ± 3.1	38.6 ± 2.8	91.9 ± 0.6	0.26 ± 0.03	0.001
	25%	Union	224	0.33 ± 0.02	31.3 ± 2.7	35.7 ± 2.3	91.1 ± 0.5	0.24 ± 0.03	0.001
		Union 2	116	0.36 ± 0.03	30.2 ± 2.2	46.2 ± 3.9	94.4 ± 0.7	0.29 ± 0.03	0.001
		Union 3	71	0.38 ± 0.02	34.6 ± 2.7	41.9 ± 1.8	92.4 ± 0.3	0.29 ± 0.02	0.001
		Intersection	17	0.33 ± 0.02	28.3 ± 1.9	39.9 ± 3.1	93.2 ± 0.6	0.25 ± 0.03	0.001
	10%	Union	94	0.39 ± 0.03	32.1 ± 2.9	48.4 ± 4.2	94.6 ± 0.5	0.31 ± 0.04	0.001
		Union 2	44	0.41 ± 0.02	33.3 ± 2.3	51.9 ± 3.2	95.1 ± 0.6	0.33 ± 0.03	0.001
		Union 3	25	0.42 ± 0.04	37.5 ± 4.3	48.2 ± 4.2	93.6 ± 0.7	0.34 ± 0.05	0.001
		Intersection	9	0.33 ± 0.02	30.9 ± 2.3	35.9 ± 1.4	91.3 ± 0.4	0.24 ± 0.02	0.001

**Table A3:** Performance of the four classification models on detection fall using *Basic data set*; basic data set exists of the 25 Foot Walk Test + 2-minute walk test + Expanded Disability Status Scale; Gaussian Naive Bayes (GNB); Decision Tree (DT); k-Nearest Neighbor (kNN); Support Vector Machine (SVM); *p*-value via permutation test.

	Thresh-Old	Combination Method	No. Features	F1	Recall (%)	Precision (%)	Specificity (%)	Kappa	*p*
**GNB**	100%	-	11	0.47 ± 0.01	57.6 ± 0.9	39.4 ± 0.4	85.7 ± 0.2	0.36 ± 0.01	0.001
	50%	Union	11	0.47 ± 0.01	57.6 ± 0.9	39.4 ± 0.4	85.7 ± 0.2	0.36 ± 0.01	0.001
		Union 2	8	0.43 ± 0.01	49.2 ± 0.8	38.1 ± 0.5	87.1 ± 0.1	0.32 ± 0.01	0.001
		Union 3	4	0.43 ± 0.01	46.9 ± 0.7	39.1 ± 0.4	88.2 ± 0.1	0.32 ± 0.01	0.001
		Intersection	1	0.05 ± 0.00	2.9 ± 0.0	42.0 ± 1.2	99.4 ± 0.0	0.04 ± 0.00	0.001
	25%	Union	7	0.46 ± 0.00	57.1 ± 0.5	37.9 ± 0.3	84.9 ± 0.1	0.35 ± 0.00	0.001
		Union 2	4	0.45 ± 0.00	48.4 ± 0.6	42.4 ± 0.4	89.4 ± 0.1	0.36 ± 0.01	0.001
		Union 3	1	0.37 ± 0.00	32.0 ± 0.0	42.6 ± 0.0	93.1 ± 0.0	0.28 ± 0.00	0.001
		Intersection	-	-	-	-	-	-	-
	10%	Union	2	0.38 ± 0.00	33.1 ± 0.0	44.7 ± 0.3	93.4 ± 0.1	0.30 ± 0.00	0.001
		Union 2	1	0.37 ± 0.00	32.0 ± 0.0	42.6 ± 0.0	93.1 ± 0.0	0.28 ± 0.00	0.001
		Union 3	1	0.37 ± 0.00	32.0 ± 0.0	42.6 ± 0.0	93.1 ± 0.0	0.28 ± 0.00	0.001
		Intersection	-	-	-	-	-	-	-
**DT**	100%	-	11	0.27 ± 0.03	20.4 ± 3.5	42.0 ± 1.8	95.5 ± 0.7	0.20 ± 0.03	0.001
	50%	Union	11	0.27 ± 0.03	20.4 ± 3.5	42.0 ± 1.8	95.5 ± 0.7	0.20 ± 0.03	0.001
		Union 2	8	0.29 ± 0.02	22.3 ± 2.1	41.7 ± 4.0	94.9 ± 0.7	0.21 ± 0.03	0.001
		Union 3	4	0.29 ± 0.04	21.7 ± 3.2	44.5 ± 4.7	95.6 ± 0.8	0.22 ± 0.04	0.001
		Intersection	1	0.00 ± 0.00	0.0 ± 0.0	0.0 ± 0.0	1.0 ± 0.0	0.00 ± 0.00	0.995
	25%	Union	7	0.23 ± 0.03	15.2 ± 2.6	44.0 ± 5.7	96.9 ± 0.5	0.17 ± 0.03	0.001
		Union 2	4	0.18 ± 0.04	12.2 ± 3.1	38.4 ± 4.1	96.9 ± 0.5	0.13 ± 0.03	0.001
		Union 3	1	0.16 ± 0.03	10.0 ± 2.1	42.5 ± 4.3	97.8 ± 0.4	0.11 ± 0.03	0.001
		Intersection	-	-	-	-	-	-	-
	10%	Union	2	0.20 ± 0.03	13.5 ± 2.3	39.8 ± 3.6	96.7 ± 0.6	0.14 ± 0.03	0.001
		Union 2	1	0.16 ± 0.03	10.0 ± 2.1	42.5 ± 4.3	97.8 ± 0.4	0.11 ± 0.03	0.001
		Union 3	1	0.16 ± 0.03	10.0 ± 2.1	42.5 ± 4.3	97.8 ± 0.4	0.11 ± 0.03	0.001
		Intersection	-	-	-	-	-	-	-
**kNN**	100%	-	11	0.26 ± 0.02	25.2 ± 2.1	26.0 ± 2.0	88.4 ± 0.5	0.14 ± 0.02	0.001
	50%	Union	11	0.26 ± 0.02	25.2 ± 2.1	26.0 ± 2.0	88.4 ± 0.5	0.14 ± 0.02	0.001
		Union 2	8	0.25 ± 0.01	25.8 ± 1.8	25.1 ± 1.3	87.6 ± 0.7	0.13 ± 0.02	0.001
		Union 3	4	0.33 ± 0.01	31.9 ± 1.3	34.2 ± 1.5	90.1 ± 0.5	0.23 ± 0.02	0.001
		Intersection	1	0.07 ± 0.08	4.7 ± 5.2	21.1 ± 14.9	98.4 ± 1.7	0.04 ± 0.05	0.001
	25%	Union	7	0.28 ± 0.02	23.4 ± 1.5	34.5 ± 2.4	92.8 ± 0.6	0.19 ± 0.02	0.001
		Union 2	4	0.24 ± 0.02	18.3 ± 1.9	37.1 ± 3.6	95.0 ± 0.6	0.17 ± 0.02	0.001
		Union 3	1	0.24 ± 0.06	21.7 ± 1.0	32.1 ± 7.3	91.7 ± 4.9	0.15 ± 0.04	0.001
		Intersection	-	-	-	-	-	-	-
	10%	Union	2	0.26 ± 0.03	21.0 ± 3.6	34.8 ± 7.4	93.1 ± 2.8	0.17 ± 0.04	0.001
		Union 2	1	0.24 ± 0.06	21.7 ± 1.0	32.1 ± 7.3	91.7 ± 4.9	0.15 ± 0.04	0.001
		Union 3	1	0.24 ± 0.06	21.7 ± 1.0	32.1 ± 7.3	91.7 ± 4.9	0.15 ± 0.04	0.001
		Intersection	-	-	-	-	-	-	-
**SVM**	100%	-	11	0.26 ± 0.02	24.8 ± 2.3	27.3 ± 2.2	89.3 ± 0.6	0.15 ± 0.02	0.001
	50%	Union	11	0.26 ± 0.02	24.8 ± 2.3	27.3 ± 2.2	89.3 ± 0.6	0.15 ± 0.02	0.001
		Union 2	8	0.21 ± 0.02	15.5 ± 1.5	34.2 ± 3.4	95.2 ± 0.4	0.14 ± 0.02	0.001
		Union 3	4	0.29 ± 0.03	22.7 ± 3.0	41.9 ± 3.5	94.9 ± 0.4	0.22 ± 0.03	0.001
		Intersection	1	0.00 ± 0.00	0.0 ± 0.0	0.0 ± 0.0	1.0 ± 0.0	0.00 ± 0.00	1.000
	25%	Union	7	0.26 ± 0.02	19.8 ± 2.2	36.9 ± 1.8	94.6 ± 0.4	0.18 ± 0.02	0.001
		Union 2	4	0.20 ± 0.02	14.0 ± 1.8	36.8 ± 3.5	96.1 ± 0.6	0.14 ± 0.02	0.001
		Union 3	1	0.08 ± 0.03	4.4 ± 1.7	37.8 ± 4.1	98.9 ± 0.3	0.05 ± 0.02	0.001
		Intersection	-	-	-	-	-	-	-
	10%	Union	2	0.17 ± 0.02	11.7 ± 1.9	34.4 ± 3.1	96.4 ± 0.6	0.11 ± 0.02	0.001
		Union 2	1	0.08 ± 0.03	4.4 ± 1.7	37.8 ± 4.1	98.9 ± 0.3	0.05 ± 0.02	0.001
		Union 3	1	0.08 ± 0.03	4.4 ± 1.7	37.8 ± 4.1	98.9 ± 0.3	0.05 ± 0.02	0.001
		Intersection	-	-	-	-	-	-	-

**Table A4:** Performance of the four classification models on detection fall using Expanded Disability Status Scale *data set*; Gaussian Naive Bayes (GNB); Decision Tree (DT); k-Nearest Neighbor (kNN); Support Vector Machine (SVM); *p*-value via permutation test.

	Thresh-Old	Combination Method	No. Features	F1	Recall (%)	Precision (%)	Specificity (%)	Kappa	*p*
**GNB**	100%	-	9	0.48 ± 0.00	64.6 ± 0.3	38.2 ± 0.2	83.1 ± 0.1	0.37 ± 0.00	0.001
	50%	Union	9	0.48 ± 0.00	64.6 ± 0.3	38.2 ± 0.2	83.1 ± 0.1	0.37 ± 0.00	0.001
		Union 2	6	0.45 ± 0.00	56.8 ± 0.5	37.2 ± 0.5	84.5 ± 0.3	0.34 ± 0.01	0.001
		Union 3	4	0.44 ± 0.01	50.5 ± 1.2	39.3 ± 0.8	87.4 ± 0.4	0.34 ± 0.01	0.001
		Intersection	1	0.05 ± 0.00	2.9 ± 0.0	42.0 ± 1.2	99.4 ± 0.0	0.04 ± 0.00	0.001
	25%	Union	4	0.44 ± 0.00	43.5 ± 0.3	43.6 ± 0.3	90.9 ± 0.0	0.35 ± 0.00	0.001
		Union 2	3	0.43 ± 0.00	42.4 ± 0.0	43.3 ± 0.1	91.0 ± 0.0	0.34 ± 0.00	0.001
		Union 3	1	0.37 ± 0.00	32.0 ± 0.0	42.6 ± 0.0	93.1 ± 0.0	0.28 ± 0.00	0.001
		Intersection	-	-	-	-	-	-	-
	10%	Union	2	0.38 ± 0.00	33.1 ± 0.0	44.7 ± 0.3	93.4 ± 0.1	0.30 ± 0.00	0.001
		Union 2	1	0.37 ± 0.00	32.0 ± 0.0	42.6 ± 0.0	93.1 ± 0.0	0.28 ± 0.00	0.001
		Union 3	1	0.37 ± 0.00	32.0 ± 0.0	42.6 ± 0.0	93.1 ± 0.0	0.28 ± 0.00	0.001
		Intersection	-	-	-	-	-	-	-
**DT**	100%	-	9	0.24 ± 0.03	16.5 ± 2.1	41.4 ± 5.0	96.2 ± 0.6	0.17 ± 0.03	0.001
	50%	Union	9	0.24 ± 0.03	16.5 ± 2.1	41.4 ± 5.0	96.2 ± 0.6	0.17 ± 0.03	0.001
		Union 2	6	0.25 ± 0.02	17.6 ± 2.1	43.8 ± 3.0	96.3 ± 0.5	0.19 ± 0.02	0.001
		Union 3	4	0.24 ± 0.02	14.3 ± 1.9	38.7 ± 2.2	96.3 ± 0.7	0.14 ± 0.02	0.001
		Intersection	1	0.00 ± 0.00	0.0 ± 0.0	0.0 ± 0.0	1.0 ± 0.0	0.00 ± 0.00	0.995
	25%	Union	4	0.20 ± 0.03	13.3 ± 2.5	43.3 ± 4.6	97.2 ± 0.5	0.15 ± 0.03	0.001
		Union 2	3	0.20 ± 0.04	13.1 ± 3.2	40.6 ± 3.8	97.0 ± 0.4	0.14 ± 0.04	0.001
		Union 3	1	0.16 ± 0.03	10.0 ± 2.1	42.5 ± 4.3	97.8 ± 0.4	0.11 ± 0.03	0.001
		Intersection	-	-	-	-	-	-	-
	10%	Union	2	0.20 ± 0.03	13.5 ± 2.3	39.8 ± 3.6	96.7 ± 0.6	0.14 ± 0.03	0.001
		Union 2	1	0.16 ± 0.03	10.0 ± 2.1	42.5 ± 4.3	97.8 ± 0.4	0.11 ± 0.03	0.001
		Union 3	1	0.16 ± 0.03	10.0 ± 2.1	42.5 ± 4.3	97.8 ± 0.4	0.11 ± 0.03	0.001
		Intersection	-	-	-	-	-	-	-
**kNN**	100%	-	9	0.28 ± 0.02	27.5 ± 1.6	29.2 ± 1.6	89.2 ± 0.5	0.17 ± 0.02	0.001
	50%	Union	9	0.28 ± 0.02	27.5 ± 1.6	29.2 ± 1.6	89.2 ± 0.5	0.17 ± 0.02	0.001
		Union 2	6	0.28 ± 0.03	24.8 ± 3.2	32.6 ± 3.9	91.7 ± 0.9	0.18 ± 0.04	0.001
		Union 3	4	0.28 ± 0.03	23.1 ± 3.1	36.4 ± 3.5	93.5 ± 0.7	0.20 ± 0.03	0.001
		Intersection	1	0.07 ± 0.08	4.7 ± 5.2	21.1 ± 14.9	98.4 ± 1.7	0.04 ± 0.05	0.001
	25%	Union	4	0.23 ± 0.02	17.3 ± 1.9	36.5 ± 2.7	95.1 ± 0.3	0.16 ± 0.02	0.001
		Union 2	3	0.25 ± 0.02	19.9 ± 1.7	33.8 ± 3.8	93.6 ± 0.9	0.16 ± 0.03	0.001
		Union 3	1	0.24 ± 0.06	21.7 ± 1.0	32.1 ± 7.3	91.7 ± 4.9	0.15 ± 0.04	0.001
		Intersection	-	-	-	-	-	-	-
	10%	Union	2	0.26 ± 0.03	21.0 ± 3.6	34.8 ± 7.4	93.1 ± 2.8	0.17 ± 0.04	0.001
		Union 2	1	0.24 ± 0.06	21.7 ± 1.0	32.1 ± 7.3	91.7 ± 4.9	0.15 ± 0.04	0.001
		Union 3	1	0.24 ± 0.06	21.7 ± 1.0	32.1 ± 7.3	91.7 ± 4.9	0.15 ± 0.04	0.001
		Intersection	-	-	-	-	-	-	-
**SVM**	100%	-	9	0.27 ± 0.02	24.1 ± 2.0	30.5 ± 2.8	91.1 ± 0.6	0.17 ± 0.03	0.001
	50%	Union	9	0.27 ± 0.02	24.1 ± 2.0	30.5 ± 2.8	91.1 ± 0.6	0.17 ± 0.03	0.001
		Union 2	6	0.24 ± 0.02	18.9 ± 2.0	33.7 ± 2.7	94.0 ± 0.5	0.16 ± 0.02	0.001
		Union 3	4	0.26 ± 0.03	19.4 ± 2.2	39.5 ± 4.0	95.2 ± 0.4	0.19 ± 0.03	0.001
		Intersection	1	0.00 ± 0.00	0.0 ± 0.0	0.0 ± 0.0	1.0 ± 0.0	0.00 ± 0.00	1.000
	25%	Union	4	0.20 ± 0.02	14.5 ± 1.3	32.7 ± 2.2	95.2 ± 0.4	0.13 ± 0.02	0.001
		Union 2	3	0.18 ± 0.03	12.7 ± 2.3	34.3 ± 3.8	96.1 ± 0.5	0.12 ± 0.03	0.001
		Union 3	1	0.08 ± 0.03	4.4 ± 1.7	37.8 ± 4.1	98.9 ± 0.3	0.05 ± 0.02	0.001
		Intersection	-	-	-	-	-	-	-
	10%	Union	2	0.17 ± 0.02	11.7 ± 1.9	34.4 ± 3.1	96.4 ± 0.6	0.11 ± 0.02	0.001
		Union 2	1	0.08 ± 0.03	4.4 ± 1.7	37.8 ± 4.1	98.9 ± 0.3	0.05 ± 0.02	0.001
		Union 3	1	0.08 ± 0.03	4.4 ± 1.7	37.8 ± 4.1	98.9 ± 0.3	0.05 ± 0.02	0.001
		Intersection	-	-	-	-	-	-	-

**Table A5:** Performance of the four classification models on detection fall using GAITRite System normal walking *data set*; Gaussian Naive Bayes (GNB); Decision Tree (DT); k-Nearest Neighbor (kNN); Support Vector Machine (SVM); *p*-value via permutation test.

	Thresh-Old	Combination Method	No. Features	F1	Recall (%)	Precision (%)	Specificity (%)	Kappa	*p*
**GNB**	100%	-	82	0.35 ± 0.01	28.7 ± 0.9	43.3 ± 0.9	94.0 ± 0.2	0.26 ± 0.01	0.001
	50%	Union	75	0.34 ± 0.01	27.7 ± 0.8	42.4 ± 1.0	93.9 ± 0.2	0.25 ± 0.01	0.001
		Union 2	52	0.35 ± 0.01	29.9 ± 0.8	43.2 ± 1.0	93.6 ± 0.2	0.27 ± 0.01	0.001
		Union 3	30	0.36 ± 0.01	31.3 ± 0.6	42.6 ± 0.7	93.2 ± 0.2	0.28 ± 0.01	0.001
		Intersection	7	0.42 ± 0.00	45.4 ± 0.6	38.4 ± 0.3	88.3 ± 0.1	0.31 ± 0.00	0.001
	25%	Union	43	0.35 ± 0.01	29.1 ± 0.7	42.5 ± 0.7	93.7 ± 0.1	0.26 ± 0.01	0.001
		Union 2	25	0.38 ± 0.01	35.1 ± 0.6	41.6 ± 0.6	92.1 ± 0.2	0.29 ± 0.01	0.001
		Union 3	13	0.40 ± 0.01	41.0 ± 0.7	38.2 ± 0.9	89.3 ± 0.3	0.29 ± 0.01	0.001
		Intersection	3	0.39 ± 0.00	37.7 ± 0.4	40.9 ± 0.4	91.2 ± 0.1	0.30 ± 0.00	0.001
	10%	Union	20	0.40 ± 0.01	40.7 ± 1.5	39.9 ± 1.1	90.1 ± 0.2	0.31 ± 0.01	0.001
		Union 2	8	0.42 ± 0.00	46.8 ± 0.6	38.0 ± 0.4	87.7 ± 0.2	0.31 ± 0.01	0.001
		Union 3	4	0.41 ± 0.01	42.3 ± 0.6	40.2 ± 0.5	89.9 ± 0.1	0.31 ± 0.01	0.001
		Intersection	-	-	-	-	-	-	-
**DT**	100%	-	82	0.24 ± 0.03	21.3 ± 3.2	28.1 ± 2.9	91.3 ± 0.6	0.14 ± 0.03	0.001
	50%	Union	75	0.25 ± 0.03	21.4 ± 3.0	29.6 ± 2.4	91.9 ± 0.7	0.15 ± 0.03	0.001
		Union 2	52	0.22 ± 0.02	17.3 ± 2.6	29.3 ± 2.4	93.3 ± 1.0	0.13 ± 0 02	0.001
		Union 3	30	0.22 ± 0.02	17.1 ± 2.5	31.2 ± 1.8	93.9 ± 0.8	0.14 ± 0.02	0.001
		Intersection	7	0.26 ± 0.03	18.8 ± 2.7	43.7 ± 3.7	96.1 ± 0.6	0.19 ± 0.03	0.001
	25%	Union	43	0.23 ± 0.03	20.4 ± 2.2	27.3 ± 4.0	91.1 ± 1.3	0.13 ± 0.03	0.001
		Union 2	25	0.22 ± 0.02	17.0 ± 1.8	31.9 ± 3.6	94.1 ± 0.9	0.14 ± 0.02	0.001
		Union 3	13	0.23 ± 0.04	16.4 ± 3.2	42.1 ± 4.6	96.4 ± 0.6	0.17 ± 0.04	0.001
		Intersection	3	0.25 ± 0.03	18.2 ± 2.8	40.1 ± 4.6	95.5 ± 1.0	0.18 ± 0.03	0.001
	10%	Union	20	0.21 ± 0.02	17.1 ± 2.5	28.4 ± 2.8	93.0 ± 1.1	0.12 ± 0.02	0.001
		Union 2	8	0.25 ± 0.03	20.1 ± 3.2	33.0 ± 4.4	93.4 ± 1.0	0.16 ± 0.04	0.001
		Union 3	4	0.25 ± 0.03	19.2 ± 3.2	35.2 ± 2.6	94.3 ± 0.7	0.17 ± 0.03	0.001
		Intersection	-	-	-	-	-	-	-
**kNN**	100%	-	82	0.20 ± 0.01	19.6 ± 0.9	20.9 ± 1.3	88.0 ± 0.7	0.08 ± 0.01	0.015
	50%	Union	75	0.20 ± 0.02	19.5 ± 1.8	21.5 ± 1.5	88.6 ± 0.4	0.08 ± 0.02	0.010
		Union 2	52	0.26 ± 0.01	24.3 ± 1.5	27.1 ± 1.5	89.4 ± 0.7	0.14 ± 0.02	0.001
		Union 3	30	0.24 ± 0.02	23.6 ± 1.8	24.8 ± 1.9	88.5 ± 0.5	0.12 ± 0.02	0.001
		Intersection	7	0.26 ± 0.02	25.1 ± 1.6	26.3 ± 1.7	88.7 ± 0.3	0.14 ± 0.02	0.002
	25%	Union	43	0.20 ± 0.01	19.3 ± 1.2	21.5 ± 1.0	88.7 ± 0.4	0.08 ± 0.01	0.005
		Union 2	25	0.25 ± 0.02	24.7 ± 1.7	25.4 ± 1.8	88.3 ± 0.8	0.13 ± 0.02	0.001
		Union 3	13	0.22 ± 0.02	20.7 ± 1.9	22.8 ± 1.4	88.8 ± 0.5	0.10 ± 0.02	0.001
		Intersection	3	0.27 ± 0.02	21.6 ± 1.8	36.3 ± 2.5	93.9 ± 0.6	0.19 ± 0.02	0.001
	10%	Union	20	0.25 ± 0.02	19.3 ± 1.4	34.0 ± 2.0	93.9 ± 0.4	0.16 ± 0.02	0.001
		Union 2	8	0.25 ± 0.02	24.0 ± 2.4	25.2 ± 2.2	88.6 ± 0.6	0.13 ± 0.03	0.001
		Union 3	4	0.30 ± 0.02	24.2 ± 1.9	39.5 ± 2.4	94.1 ± 0.4	0.22 ± 0.02	0.001
		Intersection	-	-	-	-	-	-	-
**SVM**	100%	-	82	0.21 ± 0.03	13.9 ± 2.4	41.5 ± 5.2	96.9 ± 0.3	0.15 ± 0.03	0.001
	50%	Union	75	0.24 ± 0.02	19.0 ± 1.8	33.3 ± 1.6	93.9 ± 0.4	0.16 ± 0.02	0.001
		Union 2	52	0.26 ± 0.03	18.5 ± 2.3	43.5 ± 4.2	96.1 ± 0.4	0.19 ± 0.03	0.001
		Union 3	30	0.18 ± 0.02	13.2 ± 2.1	26.9 ± 3.3	94.2 ± 0.6	0.09 ± 0.02	0.001
		Intersection	7	0.20 ± 0.03	14.8 ± 2.2	33.6 ± 5.2	95.3 ± 0.6	0.13 ± 0.03	0.001
	25%	Union	43	0.26 ± 0.04	18.5 ± 2.9	42.4 ± 4.9	96.0 ± 0.4	0.19 ± 0.04	0.001
		Union 2	25	0.18 ± 0.02	11.5 ± 1.3	47.2 ± 3.6	97.9 ± 0.1	0.14 ± 0.02	0.001
		Union 3	13	0.17 ± 0.01	15.7 ± 1.4	18.9 ± 1.2	89.1 ± 0.8	0.05 ± 0.01	0.093
		Intersection	3	0.16 ± 0.02	9.8 ± 1.5	45.2 ± 5.3	98.1 ± 0.3	0.12 ± 0.02	0.001
	10%	Union	20	0.15 ± 0.03	9.5 ± 1.9	39.5 ± 6.6	97.7 ± 0.4	0.10 ± 0.03	0.001
		Union 2	8	0.20 ± 0.02	16.4 ± 1.8	25.3 ± 2.1	92.2 ± 0.8	0.10 ± 0.02	0.002
		Union 3	4	0.19 ± 0.02	11.9 ± 1.6	44.9 ± 5.1	97.7 ± 0.2	0.14 ± 0.02	0.001
		Intersection	-	-	-	-	-	-	-

**Table A6:** Performance of the four classification models on detection fall using GAITRite System dual task walking *data set*; Gaussian Naive Bayes (GNB); Decision Tree (DT); k-Nearest Neighbor (kNN); Support Vector Machine (SVM); *p*-value via permutation test.

	Thresh-Old	Combination Method	No. Features	F1	Recall (%)	Precision (%)	Specificity (%)	Kappa	*p*
**GNB**	100%	-	82	0.33 ± 0.01	33.2 ± 1.0	32.2 ± 1.0	89.1 ± 0.4	0.22 ± 0.01	0.001
	50%	Union	67	0.34 ± 0.01	34.4 ± 0.8	33.5 ± 0.7	89.3 ± 0.2	0.23 ± 0.01	0.001
		Union 2	48	0.34 ± 0.00	35.5 ± 0.6	32.8 ± 0.4	88.6 ± 0.2	0.23 ± 0.00	0.001
		Union 3	33	0.33 ± 0.01	35.1 ± 0.9	32.0 ± 0.5	88.4 ± 0.2	0.23 ± 0.01	0.001
		Intersection	16	0.37 ± 0.00	46.1 ± 0.7	30.7 ± 0.3	83.8 ± 0.2	0.25 ± 0.00	0.001
	25%	Union	44	0.34 ± 0.01	36.2 ± 0.8	31.9 ± 0.5	88.0 ± 0.2	0.23 ± 0.01	0.001
		Union 2	25	0.36 ± 0.00	41.9 ± 0.4	31.7 ± 0.3	85.9 ± 0.2	0.24 ± 0.00	0.001
		Union 3	11	0.38 ± 0.00	44.5 ± 0.7	32.7 ± 0.4	85.7 ± 0.2	0.26 ± 0.01	0.001
		Intersection	4	0.38 ± 0.01	41.0 ± 0.8	35.7 ± 0.4	88.5 ± 0.1	0.28 ± 0.01	0.001
	10%	Union	20	0.36 ± 0.01	41.0 ± 0.7	32.0 ± 0.6	86.4 ± 0.2	0.24 ± 0 01	0.001
		Union 2	10	0.38 ± 0.00	43.8 ± 0.5	34.0 ± 0.4	86.7 ± 0.2	0.27 ± 0.00	0.001
		Union 3	2	0.31 ± 0.00	26.0 ± 0.5	39.1 ± 0.5	93.7 ± 0.1	0.23 ± 0.01	0.001
		Intersection	-	-	-	-	-	-	-
**DT**	100%	-	82	0.24 ± 0.05	19.8 ± 4.9	29.7 ± 4.2	92.8 ± 0.8	0.15 ± 0.05	0.001
	50%	Union	67	0.22 ± 0.03	17.1 ± 2.9	29.9 ± 3.7	93.7 ± 1.0	0.13 ± 0.03	0.001
		Union 2	48	0.20 ± 0.03	18.0 ± 2.8	23.7 ± 3.8	90.8 ± 1.7	0.10 ± 0.03	0.001
		Union 3	33	0.22 ± 0.02	18.0 ± 2.2	29.8 ± 3.3	93.3 ± 1.1	0.14 ± 0.02	0.001
		Intersection	16	0.18 ± 0.02	14.0 ± 2.2	24.9 ± 2.9	93.4 ± 0 8	0.09 ± 0.02	0.001
	25%	Union	44	0.18 ± 0.04	12.8 ± 3.1	31.7 ± 4.9	95.6 ± 1.3	0.11 ± 0.03	0.001
		Union 2	25	0.23 ± 0.02	19.9 ± 2.2	27.4 ± 2.6	91.7 ± 1.0	0.13 ± 0.02	0.001
		Union 3	11	0.21 ± 0.02	17.1 ± 2.2	27.3 ± 3.1	92.9 ± 0.9	0.12 ± 0.02	0.001
		Intersection	4	0.23 ± 0.03	18.4 ± 2.5	29.7 ± 3.6	93.2 ± 0.7	0.14 ± 0.03	0.001
	10%	Union	20	0.24 ± 0.04	20.1 ± 4.0	29.2 ± 4.9	92.4 ± 1.0	0.14 ± 0.04	0.001
		Union 2	10	0.23 ± 0.02	18.7 ± 2.1	29.0 ± 3.0	92.8 ± 1.0	0.13 ± 0.02	0.001
		Union 3	2	0.22 ± 0.03	17.8 ± 2.3	30.7 ± 3.2	93.7 ± 0.7	0.14 ± 0.03	0.001
		Intersection	-	-	-	-	-	-	-
**kNN**	100%	-	82	0.20 ± 0.03	13.2 ± 2.1	42.1 ± 4.1	97.2 ± 0.2	0.15 ± 0.03	0.001
	50%	Union	67	0.21 ± 0.03	20.9 ± 2.9	21.9 ± 2.5	88.4 ± 0.4	0.09 ± 0.03	0.001
		Union 2	48	0.25 ± 0.02	24.1 ± 2.6	26.4 ± 1.9	89.5 ± 0.7	0.14 ± 0.02	0.001
		Union 3	33	0.26 ± 0.02	25.4 ± 2.0	26.4 ± 1.6	88.9 ± 0.5	0.15 ± 0 02	0.001
		Intersection	16	0.21 ± 0.01	14.5 ± 1.2	35.6 ± 2.3	95.9 ± 0.4	0.14 ± 0.01	0.001
	25%	Union	44	0.25 ± 0.02	18.5 ± 1.7	40.4 ± 3.4	95.7 ± 0.5	0.18 ± 0.02	0.001
		Union 2	25	0.22 ± 0.02	20.7 ± 2.2	22.9 ± 1.9	89.1 ± 0.6	0.10 ± 0 02	0.001
		Union 3	11	0.25 ± 0.02	18.8 ± 1.5	35.6 ± 2.7	94.7 ± 0.4	0.17 ± 0.02	0.001
		Intersection	4	0.18 ± 0.02	14.3 ± 2.2	25.8 ± 2.9	93.6 ± 0.5	0.10 ± 0.03	0.001
	10%	Union	20	0.26 ± 0.03	24.8 ± 3.2	28.1 ± 2.5	90.1 ± 0.4	0.16 ± 0.03	0.001
		Union 2	10	0.28 ± 0.02	28.6 ± 2.0	27.1 ± 1.2	88.0 ± 0.4	0.16 ± 0.02	0.001
		Union 3	2	0.27 ± 0.02	25.9 ± 2.5	27.3 ± 2.2	89.2 ± 0.5	0.15 ± 0.03	0.001
		Intersection	-	-	-	-	-	-	-
**SVM**	100%	-	82	0.20 ± 0.02	16.3 ± 2.2	26.0 ± 2.4	92.8 ± 0.6	0.11 ± 0.02	0.001
	50%	Union	67	0.22 ± 0.02	17.7 ± 2.5	28.9 ± 2.5	93.2 ± 0.5	0.13 ± 0.02	0.001
		Union 2	48	0.23 ± 0.03	16.0 ± 2.0	40.4 ± 4.6	96.3 ± 0.3	0.16 ± 0.03	0.001
		Union 3	33	0.19 ± 0.02	12.1 ± 1.5	42.5 ± 6.9	97.4 ± 0.6	0.14 ± 0.03	0.001
		Intersection	16	0.20 ± 0.03	15.1 ± 2.1	28.0 ± 4.1	93.9 ± 0.5	0.11 ± 0.03	0.008
	25%	Union	44	0.24 ± 0.02	17.4 ± 1.8	37.3 ± 2.3	95.4 ± 0.4	0.17 ± 0.02	0.001
		Union 2	25	0.21 ± 0.02	14.5 ± 1.9	36.3 ± 3.4	96.0 ± 0.5	0.14 ± 0.02	0.001
		Union 3	11	0.16 ± 0.01	15.1 ± 1.3	17.7 ± 1.0	89.0 ± 0.8	0.04 ± 0.01	0.061
		Intersection	4	0.13 ± 0.01	7.8 ± 1.0	36.1 ± 3.4	97.8 ± 0.2	0.08 ± 0.01	0.001
	10%	Union	20	0.24 ± 0.02	16.8 ± 1.7	41.0 ± 2.7	96.2 ± 0.5	0.17 ± 0.02	0.001
		Union 2	10	0.21 ± 0.02	14.6 ± 1.5	37.3 ± 2.2	96.2 ± 0.3	0.14 ± 0.02	0.001
		Union 3	2	0.14 ± 0.02	8.4 ± 1.5	47.6 ± 4.1	98.6 ± 0.2	0.11 ± 0.02	0.001
		Intersection	-	-	-	-	-	-	-

**Table A7:** Performance of the four classification models on detection fall using Mobility Lab Gait normal walking *data set*; Gaussian Naive Bayes (GNB); Decision Tree (DT); k-Nearest Neighbor (kNN); Support Vector Machine (SVM); *p*-value via permutation test.

	Thresh-Old	Combination Method	No. Features	F1	Recall (%)	Precision (%)	Specificity (%)	Kappa	*p*
**GNB**	100%	-	84	0.41 ± 0.00	43.1 ± 0.7	38.2 ± 0.3	88.6 ± 0.2	0.30 ± 0.00	0.001
	50%	Union	72	0.41 ± 0.01	43.5 ± 0.9	38.7 ± 0.8	88.8 ± 0.2	0.31 ± 0.01	0.001
		Union 2	48	0.40 ± 0.01	41.0 ± 0.9	38.3 ± 0.6	89.2 ± 0.2	0.29 ± 0.01	0.001
		Union 3	36	0.39 ± 0.01	40.4 ± 0.8	38.7 ± 0.4	89.6 ± 0.1	0.29 ± 0.01	0.001
		Intersection	12	0.41 ± 0.00	44.3 ± 0.4	38.8 ± 0.6	88.6 ± 0.2	0.31 ± 0.01	0.001
	25%	Union	50	0.40 ± 0.01	42.4 ± 0.9	38.5 ± 0.6	88.9 ± 0.3	0.30 ± 0.01	0.001
		Union 2	21	0.41 ± 0.01	42.4 ± 1.0	39.8 ± 0.7	89.5 ± 0.2	0.31 ± 0.01	0.001
		Union 3	11	0.42 ± 0.00	44.8 ± 0.4	39.5 ± 0.3	88.8 ± 0.1	0.32 ± 0.00	0.001
		Intersection	2	0.36 ± 0.01	32.2 ± 1.1	39.6 ± 1.1	92.0 ± 0.1	0.26 ± 0.01	0.001
	10%	Union	22	0.42 ± 0.01	46.0 ± 0.7	38.9 ± 0.8	88.2 ± 0.3	0.32 ± 0.01	0.001
		Union 2	8	0.43 ± 0.01	42.9 ± 0.6	43.1 ± 0.7	90.8 ± 0.2	0.34 ± 0.01	0.001
		Union 3	2	0.37 ± 0.01	33.6 ± 0.8	42.4 ± 0.6	92.6 ± 0.1	0.29 ± 0.01	0.001
		Intersection	-	-	-	-	-	-	-
**DT**	100%	-	84	0.22 ± 0.04	17.1 ± 3.3	29.7 ± 4.6	93.4 ± 1.2	0.13 ± 0.04	0.001
	50%	Union	72	0.25 ± 0.03	19.2 ± 2.3	34.4 ± 3.4	94.0 ± 0.6	0.16 ± 0.03	0.001
		Union 2	48	0.23 ± 0.05	17.3 ± 5.2	36.8 ± 3.0	95.2 ± 1.0	0.16 ± 0.04	0.001
		Union 3	36	0.24 ± 0.03	21.4 ± 3.0	26.8 ± 3.3	90.4 ± 1.3	0.13 ± 0.03	0.001
		Intersection	12	0.23 ± 0.04	15.6 ± 3.6	42.5 ± 6.1	96.6 ± 0.6	0.16 ± 0.04	0.001
	25%	Union	50	0.19 ± 0.05	13.5 ± 4.4	36.5 ± 4.7	96.2 ± 1.2	0.13 ± 0.04	0.001
		Union 2	21	0.23 ± 0.03	16.8 ± 3.3	39.7 ± 2.6	95.8 ± 0.8	0.17 ± 0.03	0.001
		Union 3	11	0.26 ± 0.03	19.6 ± 2.9	37.2 ± 3.2	94.6 ± 0.5	0.18 ± 0.03	0.001
		Intersection	2	0.23 ± 0.02	16.0 ± 1.3	43.1 ± 3.4	96.5 ± 0.6	0.17 ± 0.02	0.001
	10%	Union	22	0.23 ± 0.07	17.4 ± 7.1	37.6 ± 5.5	95.4 ± 1.5	0.16 ± 0.06	0.001
		Union 2	8	0.24 ± 0.03	16.4 ± 2.6	42.5 ± 3.9	96.4 ± 0.5	0.17 ± 0.03	0.001
		Union 3	2	0.24 ± 0.03	17.0 ± 2.2	43.1 ± 3.5	96.3 ± 0.5	0.18 ± 0.02	0.001
		Intersection	-	-	-	-	-	-	-
**kNN**	100%	-	84	0.22 ± 0.02	21.6 ± 2.8	22.8 ± 2.1	88.2 ± 0.6	0.10 ± 0.03	0.001
	50%	Union	72	0.25 ± 0.03	25.0 ± 3.0	24.5 ± 2.5	87.4 ± 0.7	0.12 ± 0.03	0.006
		Union 2	48	0.26 ± 0.01	26.3 ± 1.7	26.5 ± 1.4	88.1 ± 0.4	0.14 ± 0.02	0.001
		Union 3	36	0.26 ± 0 01	25.2 ± 1.2	26.3 ± 1.4	88.5 ± 0.7	0.14 ± 0.01	0.023
		Intersection	12	0.22 ± 0.02	21.3 ± 2.4	23.0 ± 2.1	88.4 ± 0.5	0.10 ± 0.02	0.005
	25%	Union	50	0.24 ± 0.02	22.0 ± 1.8	27.1 ± 1.4	90.4 ± 0.4	0.13 ± 0.02	0.002
		Union 2	21	0.21 ± 0.01	20.5 ± 1.3	21.9 ± 1.3	88.1 ± 0.6	0.09 ± 0.01	0.021
		Union 3	11	0.21 ± 0.02	15.6 ± 1.9	32.2 ± 3.9	94.6 ± 0.5	0.13 ± 0.03	0.001
		Intersection	2	0.21 ± 0.02	21.0 ± 2.5	21.5 ± 2.0	87.5 ± 0.7	0.09 ± 0.02	0.019
	10%	Union	22	0.31 ± 0.02	30.1 ± 1.9	32.2 ± 2.1	89.6 ± 0.8	0.20 ± 0.02	0.001
		Union 2	8	0.26 ± 0.03	25.1 ± 3.1	28.1 ± 2.5	89.5 ± 0.6	0.15 ± 0.03	0.001
		Union 3	2	0.21 ± 0.03	17.1 ± 2.6	28.9 ± 3.5	93.2 ± 0.6	0.12 ± 0.03	0.001
		Intersection	-	-	-	-	-	-	-
**SVM**	100%	-	84	0.17 ± 0.02	11.8 ± 1.5	29.2 ± 2.2	95.4 ± 0.4	0.09 ± 0.02	0.001
	50%	Union	72	0.20 ± 0.02	17.3 ± 1.6	24.2 ± 2.3	91.1 ± 0.8	0.10 ± 0.02	0.001
		Union 2	48	0.27 ± 0.01	21.1 ± 1.3	36.9 ± 2.5	94.1 ± 0.6	0.18 ± 0.02	0.001
		Union 3	36	0.22 ± 0.02	15.5 ± 1.9	36.4 ± 3.5	95.6 ± 0.3	0.15 ± 0.03	0.001
		Intersection	12	0.22 ± 0.02	18.7 ± 2.5	26.2 ± 2.8	91.4 ± 1.0	0.11 ± 0.03	0.001
	25%	Union	50	0.23 ± 0.04	20.8 ± 3.4	27.1 ± 3.6	90.9 ± 0.5	0.13 ± 0.04	0.001
		Union 2	21	0.21 ± 0.02	17.9 ± 2.3	24.6 ± 1.7	91.1 ± 0.5	0.10 ± 0.02	0.002
		Union 3	11	0.27 ± 0.02	21.3 ± 1.9	36.9 ± 2.3	94.1 ± 0.6	0.19 ± 0.02	0.001
		Intersection	2	0.19 ± 0.02	11.8 ± 1.6	55.5 ± 4.3	98.5 ± 0.3	0.15 ± 0.02	0.001
	10%	Union	22	0.28 ± 0.02	20.1 ± 1.9	46.9 ± 4.1	96.3 ± 0.6	0.22 ± 0.02	0.001
		Union 2	8	0.17 ± 0.03	11.2 ± 2.2	36.2 ± 4.0	96.8 ± 0.3	0.11 ± 0.03	0.001
		Union 3	2	0.06 ± 0.02	3.1 ± 0.9	44.5 ± 7.1	99.3 ± 0.3	0.04 ± 0.01	0.001
		Intersection	-	-	-	-	-	-	-

**Table A8:** Performance of the four classification models on detection fall using Mobility Lab Gait dual task walking *data set*; Gaussian Naive Bayes (GNB); Decision Tree (DT); k-Nearest Neighbor (kNN); Support Vector Machine (SVM); *p*-value via permutation test.

	Thresh-Old	Combination Method	No. Features	F1	Recall (%)	Precision (%)	Specificity (%)	Kappa	*p*
**GNB**	100%	-	84	0.36 ± 0.01	44.9 ± 0.9	30.2 ± 0.5	83.6 ± 0.3	0.24 ± 0.01	0.001
	50%	Union	61	0.37 ± 0.00	45.5 ± 0.5	31.2 ± 0.4	84.1 ± 0.1	0.25 ± 0.00	0.001
		Union 2	45	0.35 ± 0.00	43.1 ± 0.4	29.9 ± 0.4	83.9 ± 0.3	0.23 ± 0.00	0.001
		Union 3	39	0.36 ± 0.01	43.7 ± 0.7	30.1 ± 0.5	83.9 ± 0.3	0.23 ± 0.01	0.001
		Intersection	23	0.36 ± 0.00	42.5 ± 0.6	31.5 ± 0.4	85.3 ± 0.2	0.24 ± 0.01	0.001
	25%	Union	44	0.36 ± 0.00	44.2 ± 0.5	30.9 ± 0.5	84.3 ± 0.3	0.24 ± 0.01	0.001
		Union 2	23	0.38 ± 0.00	44.9 ± 0.5	32.8 ± 0.3	85.4 ± 0.2	0.26 ± 0.00	0.001
		Union 3	13	0.39 ± 0.01	45.3 ± 0.6	33.7 ± 0.5	85.9 ± 0.2	0.27 ± 0.01	0.001
		Intersection	4	0.39 ± 0.00	40.7 ± 0.6	37.4 ± 0.5	89.2 ± 0.1	0.29 ± 0.01	0.001
	10%	Union	21	0.37 ± 0.01	43.6 ± 0.8	31.6 ± 0.5	85.0 ± 0.2	0.25 ± 0.01	0.001
		Union 2	7	0.37 ± 0.00	42.1 ± 0.5	33.2 ± 0.4	86.6 ± 0.1	0.26 ± 0.00	0.001
		Union 3	3	0.38 ± 0.01	36.0 ± 0.7	39.4 ± 0.6	91.2 ± 0.1	0.28 ± 0.01	0.001
		Intersection	1	0.20 ± 0.01	12.7 ± 0.7	42.8 ± 2.4	97.3 ± 0.2	0.14 ± 0.01	0.001
**DT**	100%	-	84	0.21 ± 0.03	16.9 ± 2.4	26.9 ± 4.1	92.6 ± 1.0	0.11 ± 0.03	0.001
	50%	Union	61	0.18 ± 0.04	13.9 ± 3.5	26.9 ± 4.6	94.0 ± 1.3	0.10 ± 0.04	0.001
		Union 2	45	0.20 ± 0.03	15.6 ± 2.8	30.3 ± 3.7	94.3 ± 1.0	0.12 ± 0.03	0.001
		Union 3	39	0.21 ± 0.02	16.5 ± 2.4	29.5 ± 4.2	93.6 ± 1.5	0.12 ± 0.03	0.001
		Intersection	23	0.21 ± 0.05	16.8 ± 5.2	28.6 ± 5.4	93.3 ± 1.7	0.12 ± 0.05	0.001
	25%	Union	44	0.21 ± 0.03	20.3 ± 4.0	22.3 ± 2.9	88.8 ± 1.4	0.09 ± 0.03	0.001
		Union 2	23	0.23 ± 0.03	17.4 ± 2.8	34.0 ± 5.9	94.5 ± 1.2	0.15 ± 0.04	0.001
		Union 3	13	0.20 ± 0.02	15.0 ± 2.0	32.2 ± 3.1	95.0 ± 0.7	0.13 ± 0.02	0.001
		Intersection	4	0.21 ± 0.04	15.9 ± 3.3	31.6 ± 5.2	94.5 ± 0.9	0.13 ± 0.04	0.001
	10%	Union	21	0.22 ± 0.03	17.8 ± 3.8	28.1 ± 2.7	92.8 ± 1.1	0.12 ± 0.03	0.001
		Union 2	7	0.25 ± 0.03	18.2 ± 2.8	38.0 ± 4.5	95.3 ± 0.7	0.17 ± 0.03	0.001
		Union 3	3	0.28 ± 0.03	22.4 ± 3.0	36.5 ± 3.5	93.8 ± 0.4	0.19 ± 0.03	0.001
		Intersection	1	0.18 ± 0.03	11.8 ± 2.6	35.7 ± 5.2	96.6 ± 0.6	0.12 ± 0.03	0.001
**kNN**	100%	-	84	0.18 ± 0.02	17.4 ± 2.0	18.8 ± 1.8	88.1 ± 0.9	0.06 ± 0.02	0.045
	50%	Union	61	0.20 ± 0.02	18.3 ± 2.8	21.6 ± 2.0	89.5 ± 0.7	0.08 ± 0.02	0.009
		Union 2	45	0.26 ± 0.02	25.3 ± 2.2	26.4 ± 2.0	88.8 ± 0.9	0.14 ± 0.02	0.001
		Union 3	39	0.18 ± 0.01	17.4 ± 1.4	19.5 ± 1.4	88.6 ± 0.7	0.06 ± 0.01	0.007
		Intersection	23	0.22 ± 0.02	16.5 ± 1.8	33.4 ± 4.0	94.7 ± 0.7	0.14 ± 0.03	0.001
	25%	Union	44	0.25 ± 0.02	24.3 ± 2.2	25.9 ± 1.7	89.0 ± 0.4	0.14 ± 0.02	0.001
		Union 2	23	0.21 ± 0.02	19.1 ± 2.5	22.9 ± 2.3	89.8 ± 0.4	0.10 ± 0.03	0.006
		Union 3	13	0.20 ± 0.02	18.6 ± 1.5	22.1 ± 1.8	89.6 ± 0.5	0.09 ± 0.02	0.028
		Intersection	4	0.21 ± 0.02	19.8 ± 1.9	23.5 ± 2.0	89.8 ± 0.6	0.10 ± 0.02	0.003
	10%	Union	21	0.24 ± 0.02	18.5 ± 1.6	35.6 ± 3.5	94.7 ± 0.5	0.16 ± 0.02	0.001
		Union 2	7	0.22 ± 0.01	20.4 ± 1.5	22.9 ± 1.5	89.1 ± 0.5	0.10 ± 0.02	0.038
		Union 3	3	0.24 ± 0.02	23.1 ± 2.3	25.4 ± 2.5	89.2 ± 0.9	0.13 ± 0.03	0.001
		Intersection	1	0.19 ± 0 03	16.8 ± 2.6	22.4 ± 3.5	90.8 ± 0.7	0.08 ± 0.03	0.005
**SVM**	100%	-	84	0.19 ± 0.02	15.0 ± 1.8	24.9 ± 3.1	92.8 ± 1.0	0.09 ± 0.02	0.001
	50%	Union	61	0.25 ± 0.02	21.9 ± 2.3	28.6 ± 1.8	91.3 ± 0 6	0.15 ± 0.02	0.001
		Union 2	45	0.22 ± 0.03	18.5 ± 2.5	27.4 ± 3.3	92.2 ± 0.6	0.12 ± 0.03	0.001
		Union 3	39	0.21 ± 0.03	15.9 ± 2.3	29.2 ± 3.1	93.9 ± 0.4	0.12 ± 0.03	0.002
		Intersection	23	0.23 ± 0.03	20.4 ± 2.5	25.6 ± 2.6	90.6 ± 0.4	0.12 ± 0.03	0.001
	25%	Union	44	0.26 ± 0.02	22.5 ± 2.0	29.7 ± 2.9	91.5 ± 0.6	0.16 ± 0.03	0.001
		Union 2	23	0.19 ± 0.02	13.5 ± 1.7	30.2 ± 3.8	95.0 ± 0.4	0.11 ± 0.02	0.001
		Union 3	13	0.21 ± 0.02	17.3 ± 2.4	28.1 ± 1.9	93.0 ± 0.8	0.12 ± 0.02	0.001
		Intersection	4	0.21 ± 0.02	18.9 ± 1.9	24.1 ± 2.2	90.6 ± 0.8	0.10 ± 0.02	0.002
	10%	Union	21	0.22 ± 0.03	17.2 ± 2.3	31.5 ± 3.6	94.1 ± 0.5	0.14 ± 0.03	0.001
		Union 2	7	0.19 ± 0.03	12.7 ± 2.0	35.4 ± 4.6	96.3 ± 0.5	0.12 ± 0.03	0.001
		Union 3	3	0.20 ± 0.03	14.6 ± 2.7	30.0 ± 3.4	94.6 ± 0.5	0.12 ± 0.03	0.015
		Intersection	1	0.03 ± 0 02	1.6 ± 1.1	37.0 ± 16.9	99.6 ± 0.1	0.02 ± 0.02	0.024

**Table A9:** Performance of the four classification models on detection fall using Mobility Lab Romberg stance with open eyes *data set*; Gaussian Naive Bayes (GNB); Decision Tree (DT); k-Nearest Neighbor (kNN); Support Vector Machine (SVM); *p*-value via permutation test.

	Thresh-Old	Combination Method	No. Features	F1	Recall (%)	Precision (%)	Specificity (%)	Kappa	*p*
**GNB**	100%	-	32	0.26 ± 0.01	20.3 ± 0.8	36.6 ± 1.8	94.4 ± 0.2	0.18 ± 0.01	0.001
	50%	Union	31	0.26 ± 0.01	20.2 ± 0.9	36.5 ± 1.6	94.4 ± 0.2	0.18 ± 0.01	0.001
		Union 2	22	0.26 ± 0.02	19.3 ± 1.3	38.8 ± 2.1	95.2 ± 0.2	0.18 ± 0.02	0.001
		Union 3	11	0.22 ± 0.01	15.7 ± 1.0	38.5 ± 1.6	96.0 ± 0.2	0.16 ± 0.01	0.001
		Intersection	-	-	-	-	-	-	-
	25%	Union	21	0.24 ± 0.01	17.5 ± 0.7	36.5 ± 1.3	95.2 ± 0.2	0.16 ± 0.01	0.001
		Union 2	9	0.21 ± 0.01	14.1 ± 0.7	38.6 ± 1.3	96.4 ± 0.2	0.14 ± 0.01	0.001
		Union 3	2	0.18 ± 0.01	11.0 ± 0.7	42.8 ± 1.7	97.7 ± 0.1	0.13 ± 0.01	0.001
		Intersection	-	-	-	-	-	-	-
	10%	Union	8	0.21 ± 0.01	14.8 ± 0.8	38.9 ± 1.9	96.3 ± 0.2	0.15 ± 0.01	0.001
		Union 2	3	0.22 ± 0.01	15.5 ± 1.0	40.9 ± 1.2	96.4 ± 0.2	0.16 ± 0.01	0.001
		Union 3	1	0.17 ± 0.01	11.0 ± 0.6	41.9 ± 1.9	97.6 ± 0.1	0.12 ± 0.01	0.001
		Intersection	-	-	-	-	-	-	-
**DT**	100%	-	32	0.21 ± 0.04	16.4 ± 3.9	31.3 ± 4.1	94.3 ± 0.9	0.13 ± 0.04	0.001
	50%	Union	31	0.20 ± 0.04	15.8 ± 3.2	29.5 ± 4.5	93.9 ± 1.1	0.12 ± 0.04	0.001
		Union 2	22	0.21 ± 0.03	17.0 ± 2.8	26.9 ± 4.0	92.7 ± 0.6	0.11 ± 0.04	0.001
		Union 3	11	0.22 ± 0.02	18.6 ± 1.8	27.3 ± 2.3	92.1 ± 0.5	0.12 ± 0.02	0.001
		Intersection	-	-	-	-	-	-	-
	25%	Union	21	0.22 ± 0.03	15.8 ± 2.5	34.4 ± 3.7	95.2 ± 0.8	0.14 ± 0.03	0.001
		Union 2	9	0.21 ± 0.02	15.5 ± 1.8	33.5 ± 3.5	95.0 ± 1.0	0.14 ± 0.02	0.001
		Union 3	2	0.25 ± 0.03	19.4 ± 2.9	34.7 ± 3.1	94.2 ± 0.7	0.17 ± 0.03	0.001
		Intersection	-	-	-	-	-	-	-
	10%	Union	8	0.21 ± 0.02	17.5 ± 1.9	27.2 ± 2.1	92.5 ± 1.0	0.12 ± 0.02	0.001
		Union 2	3	0.23 ± 0.02	17.5 ± 1.7	33.4 ± 4.2	94.4 ± 1.0	0.15 ± 0.02	0.001
		Union 3	1	0.24 ± 0.02	15.7 ± 1.7	52.2 ± 4.2	97.7 ± 0.5	0.19 ± 0.02	0.001
		Intersection	-	-	-	-	-	-	-
**kNN**	100%	-	32	0.23 ± 0.01	24.2 ± 0.9	22.0 ± 1.2	86.4 ± 0.6	0.10 ± 0.01	0.001
	50%	Union	31	0.24 ± 0.03	18.1 ± 2.2	34.8 ± 3.2	94.6 ± 0.4	0.16 ± 0.03	0.001
		Union 2	22	0.24 ± 0.01	23.0 ± 1.5	24.8 ± 1.2	88.9 ± 0.6	0.12 ± 0.01	0.001
		Union 3	11	0.25 ± 0.03	25.0 ± 2.8	24.4 ± 2.3	87.7 ± 0.5	0.13 ± 0.03	0.001
		Intersection	-	-	-	-	-	-	-
	25%	Union	21	0.20 ± 0.02	13.4 ± 1.4	38.8 ± 3.5	96.6 ± 0.3	0.14 ± 0.02	0.001
		Union 2	9	0.27 ± 0.02	26.7 ± 2.2	26.4 ± 2.0	88.2 ± 0.4	0.15 ± 0.02	0.001
		Union 3	2	0.29 ± 0.02	24.8 ± 1.5	35.0 ± 1.6	92.7 ± 0.3	0.20 ± 0.02	0.001
		Intersection	-	-	-	-	-	-	-
	10%	Union	8	0.25 ± 0.02	19.1 ± 1.9	34.7 ± 2.8	94.3 ± 0.4	0.16 ± 0.02	0.001
		Union 2	3	0.22 ± 0.01	21.9 ± 1.1	22.2 ± 1.5	87.8 ± 0.6	0.10 ± 0.02	0.002
		Union 3	1	0.23 ± 0.03	14.9 ± 1.9	53.4 ± 4.2	97.9 ± 0.2	0.18 ± 0.03	0.001
		Intersection	-	-	-	-	-	-	-
**SVM**	100%	-	32	0.18 ± 0.02	15.6 ± 1.6	20.4 ± 2.3	90.2 ± 0.6	0.07 ± 0.02	0.057
	50%	Union	31	0.14 ± 0.02	9.6 ± 1.3	28.8 ± 3.8	96.2 ± 0.4	0.08 ± 0.02	0.002
		Union 2	22	0.20 ± 0.01	13.5 ± 1.0	35.8 ± 4.4	96.1 ± 0.6	0.13 ± 0.02	0.001
		Union 3	11	0.19 ± 0.03	13.5 ± 2.7	33.5 ± 3.3	95.8 ± 0.3	0.12 ± 0.03	0.001
		Intersection	-	-	-	-	-	-	-
	25%	Union	21	0.18 ± 0.01	15.8 ± 1.3	21.8 ± 1.7	90.9 ± 0.9	0.08 ± 0.01	0.007
		Union 2	9	0.18 ± 0.03	13.8 ± 2.4	25.5 ± 4.3	93.6 ± 0.6	0.09 ± 0.03	0.001
		Union 3	2	0.13 ± 0.03	7.6 ± 1.8	39.9 ± 7.2	98.2 ± 0.3	0.09 ± 0.03	0.005
		Intersection	-	-	-	-	-	-	-
	10%	Union	8	0.18 ± 0.02	13.8 ± 1.7	27.9 ± 3.5	94.3 ± 0.6	0.10 ± 0.02	0.001
		Union 2	3	0.09 ± 0.02	5.2 ± 1.5	27.7 ± 5.2	97.9 ± 0.3	0.05 ± 0.02	0.006
		Union 3	1	0.09 ± 0.02	4.9 ± 1.3	66.5 ± 12.9	99.6 ± 0.4	0.07 ± 0.02	0.001
		Intersection	-	-	-	-	-	-	-

**Table A10:** Performance of the four classification models on detection fall using Mobility Lab Romberg stance with closed eyes (*data set*; Gaussian Naive Bayes (GNB); Decision Tree (DT); k-Nearest Neighbor (kNN); Support Vector Machine (SVM); *p*-value via permutation test.

	Thresh-Old	Combination Method	No. Features	F1	Recall (%)	Precision (%)	Specificity (%)	Kappa	*p*
**GNB**	100%	-	32	0.27 ± 0.01	21.5 ± 0.8	37.7 ± 1.2	94.4 ± 0.1	0.19 ± 0.01	0.001
	50%	Union	30	0.28 ± 0.01	21.7 ± 0.9	38.6 ± 1.1	94.6 ± 0.1	0.20 ± 0.01	0.001
		Union 2	22	0.27 ± 0.01	20.1 ± 0.9	39.9 ± 1.4	95.2 ± 0.1	0.19 ± 0.01	0.001
		Union 3	10	0.26 ± 0.01	18.5 ± 0.3	44.3 ± 2.1	96.3 ± 0.3	0.20 ± 0.01	0.001
		Intersection	2	0.29 ± 0.00	20.9 ± 0.4	45.3 ± 0.4	96.0 ± 0.1	0.22 ± 0.00	0.001
	25%	Union	22	0.30 ± 0.01	23.4 ± 0.9	40.8 ± 1.3	94.6 ± 0.2	0.22 ± 0.01	0.001
		Union 2	9	0.26 ± 0.01	18.5 ± 0.7	46.3 ± 2.2	96.6 ± 0.3	0.20 ± 0.01	0.001
		Union 3	1	0.26 ± 0.01	18.5 ± 0.3	44.3 ± 2.1	96.3 ± 0.3	0.20 ± 0.01	0.001
		Intersection	-	-	-	-	-	-	-
	10%	Union	10	0.26 ± 0.01	19.1 ± 0.7	41.6 ± 1.2	95.8 ± 0.1	0.19 ± 0.01	0.001
		Union 2	2	0.12 ± 0.01	7.2 ± 0.7	46.8 ± 2.6	98.7 ± 0.1	0.09 ± 0.01	0.001
		Union 3	-	-	-	-	-	-	-
		Intersection	-	-	-	-	-	-	-
**DT**	100%	-	32	0.21 ± 0.04	18.1 ± 4.2	24.6 ± 2.7	91.3 ± 1.0	0.11 ± 0.03	0.001
	50%	Union	30	0.22 ± 0.04	15.4 ± 3.5	37.2 ± 4.8	95.9 ± 0.7	0.15 ± 0.04	0.001
		Union 2	22	0.22 ± 0.04	17.0 ± 3.4	31.6 ± 3.8	94.2 ± 0.8	0.14 ± 0.03	0.001
		Union 3	10	0.23 ± 0.04	16.5 ± 3.5	38.1 ± 5.3	95.8 ± 0.7	0.16 ± 0.04	0.001
		Intersection	2	0.20 ± 0.02	15.1 ± 2.1	30.2 ± 2.6	94.5 ± 0.7	0.12 ± 0.02	0.001
	25%	Union	22	0.18 ± 0.04	13.8 ± 3.6	25.6 ± 4.1	93.8 ± 0.9	0.09 ± 0.04	0.001
		Union 2	9	0.20 ± 0.03	15.4 ± 2.7	28.8 ± 3.6	94.0 ± 0.5	0.12 ± 0.03	0.001
		Union 3	1	0.23 ± 0.04	16.5 ± 3.5	38.1 ± 5.3	95.8 ± 0.7	0.16 ± 0.04	0.001
		Intersection	-	-	-	-	-	-	-
	10%	Union	10	0.19 ± 0.04	13.6 ± 3.0	34.2 ± 5.3	95.9 ± 0.8	0.13 ± 0.04	0.001
		Union 2	2	0.24 ± 0.02	16.6 ± 1.3	43.9 ± 2.6	96.6 ± 0.3	0.18 ± 0.02	0.001
		Union 3	-	-	-	-	-	-	-
		Intersection	-	-	-	-	-	-	-
**kNN**	100%	-	32	0.17 ± 0.02	12.9 ± 1.6	24.2 ± 2.8	93.6 ± 0.3	0.08 ± 0.02	0.001
	50%	Union	30	0.16 ± 0.02	12.0 ± 1.4	23.4 ± 2.3	93.8 ± 0.4	0.07 ± 0.02	0.001
		Union 2	22	0.20 ± 0.02	20.5 ± 2.4	19.8 ± 1.7	86.9 ± 0.7	0.07 ± 0.02	0.002
		Union 3	10	0.21 ± 0.02	16.6 ± 2.0	28.8 ± 2.3	93.5 ± 0.4	0.12 ± 0.02	0.001
		Intersection	2	0.22 ± 0.02	22.2 ± 2.3	21.5 ± 2.2	87.2 ± 0.7	0.09 ± 0.03	0.006
	25%	Union	22	0.24 ± 0.02	22.7 ± 1.7	24.5 ± 1.7	89.0 ± 0.4	0.12 ± 0.02	0.002
		Union 2	9	0.23 ± 0.02	22.2 ± 2.0	24.5 ± 1.7	89.2 ± 0.4	0.12 ± 0 02	0.001
		Union 3	1	0.21 ± 0.02	16.6 ± 2.0	28.8 ± 2.3	93.5 ± 0.4	0.12 ± 0.02	0.005
		Intersection	-	-	-	-	-	-	-
	10%	Union	10	0.24 ± 0.01	24.0 ± 1.3	24.8 ± 1.0	88.5 ± 0.5	0.13 ± 0.01	0.001
		Union 2	2	0.22 ± 0.03	17.3 ± 2.2	29.7 ± 4.2	93.5 ± 0.6	0.13 ± 0.03	0.001
		Union 3	-	-	-	-	-	-	-
		Intersection	-	-	-	-	-	-	-
**SVM**	100%	-	32	0.18 ± 0.02	16.0 ± 1.8	20.8 ± 2.3	90.4 ± 0.7	0.07 ± 0.02	0.002
	50%	Union	30	0.18 ± 0.01	15.6 ± 1.5	20.6 ± 1.7	90.5 ± 0.6	0.07 ± 0.02	0.007
		Union 2	22	0.19 ± 0.02	14.1 ± 1.6	29.8 ± 3.1	94.7 ± 0.6	0.11 ± 0.02	0.001
		Union 3	10	0.15 ± 0.02	10.2 ± 1.2	27.4 ± 2.7	95.7 ± 0.3	0.08 ± 0.02	0.001
		Intersection	2	0.10 ± 0.01	5.6 ± 0.8	37.0 ± 2.2	98.5 ± 0.1	0.06 ± 0.01	0.001
	25%	Union	22	0.18 ± 0.02	15.8 ± 2.1	21.0 ± 2.5	90.6 ± 0.6	0.07 ± 0.02	0.002
		Union 2	9	0.19 ± 0.02	16.0 ± 1.6	24.5 ± 2.3	92.2 ± 0.6	0.10 ± 0.02	0.001
		Union 3	1	0.15 ± 0.02	10.2 ± 1.2	27.4 ± 2.7	95.7 ± 0.3	0.08 ± 0.02	0.001
		Intersection	-	-	-	-	-	-	-
	10%	Union	10	0.18 ± 0.03	13.2 ± 1.9	30.0 ± 4.3	95.1 ± 0.4	0.11 ± 0.03	0.001
		Union 2	2	0.10 ± 0.02	5.6 ± 0.9	50.6 ± 5.8	99.1 ± 0.2	0.08 ± 0.01	0.001
		Union 3	-	-	-	-	-	-	-
		Intersection	-	-	-	-	-	-	-

**Table A11:** Performance of the four classification models on detection fall using Twelve Item Multiple Sclerosis Walking Scale *data set*; Gaussian Naive Bayes (GNB); Decision Tree (DT); k-Nearest Neighbor (kNN); Support Vector Machine (SVM); *p*-value via permutation test.

	Thresh-Old	Combination Method	No. Features	F1	Recall (%)	Precision (%)	Specificity (%)	Kappa	*p*
**GNB**	100%	-	12	0.50 ± 0.00	70.9 ± 0.4	39.1 ± 0.2	82.3 ± 0.1	0.40 ± 0.00	0.001
	50%	Union	10	0.51 ± 0.00	71.4 ± 0.3	39.5 ± 0.1	82.5 ± 0.1	0.40 ± 0.00	0.001
		Union 2	8	0.52 ± 0.00	70.8 ± 0.4	41.0 ± 0.2	83.6 ± 0.1	0.42 ± 0.00	0.001
		Union 3	5	0.52 ± 0.00	67.5 ± 0.5	42.3 ± 0.3	85.2 ± 0.2	0.42 ± 0.00	0.001
		Intersection	1	0.30 ± 0.05	23.8 ± 5.5	44.2 ± 2.7	95.1 ± 1.4	0.23 ± 0.04	0.001
	25%	Union	7	0.53 ± 0.00	71.6 ± 0.4	41.8 ± 0.2	84.0 ± 0.1	0.43 ± 0.00	0.001
		Union 2	3	0.54 ± 0.00	65.9 ± 0.0	45.4 ± 0.1	87.3 ± 0.0	0.45 ± 0.00	0.001
		Union 3	1	0.30 ± 0.05	23.8 ± 5.5	44.2 ± 2.7	95.1 ± 1.4	0.23 ± 0.04	0.001
		Intersection	1	0.30 ± 0.05	23.8 ± 5.5	44.2 ± 2.7	95.1 ± 1.4	0.23 ± 0.04	0.001
	10%	Union	2	0.48 ± 0.00	50.2 ± 0.3	46.2 ± 0.2	90.6 ± 0.1	0.39 ± 0.00	0.001
		Union 2	1	0.30 ± 0.05	23.8 ± 5.5	44.2 ± 2.7	95.1 ± 1.4	0.23 ± 0.04	0.001
		Union 3	1	0.30 ± 0.05	23.8 ± 5.5	44.2 ± 2.7	95.1 ± 1.4	0.23 ± 0.04	0.001
		Intersection	-	-	-	-	-	-	-
**DT**	100%	-	12	0.33 ± 0.03	24.4 ± 1.9	49.8 ± 6.3	96.0 ± 1.0	0.26 ± 0.03	0.001
	50%	Union	10	0.30 ± 0.07	23.3 ± 6.8	45.2 ± 5.0	95.5 ± 1.3	0.23 ± 0.06	0.001
		Union 2	8	0.33 ± 0.04	26.6 ± 4.9	42.8 ± 3.2	94.3 ± 1.1	0.25 ± 0.04	0.001
		Union 3	5	0.39 ± 0.04	31.1 ± 4.3	50.9 ± 3.8	95.2 ± 0.6	0.31 ± 0.04	0.001
		Intersection	1	0.01 ± 0.02	0.5 ± 1.1	6.3 ± 13.5	99.8 ± 0.3	0.01 ± 0.01	1.000
	25%	Union	7	0.33 ± 0.08	26.7 ± 8.5	43.6 ± 4.2	94.6 ± 1.6	0.25 ± 0.07	0.001
		Union 2	3	0.35 ± 0.03	28.3 ± 3.8	45.8 ± 2.6	94.6 ± 0.7	0.27 ± 0.03	0.001
		Union 3	1	0.01 ± 0.02	0.5 ± 1.1	6.3 ± 13.5	99.8 ± 0.3	0.01 ± 0.01	1.000
		Intersection	1	0.01 ± 0.02	0.5 ± 1.1	6.3 ± 13.5	99.8 ± 0.3	0.01 ± 0.01	1.000
	10%	Union	2	0.22 ± 0.04	14.1 ± 3.1	55.7 ± 7.1	98.1 ± 0.9	0.18 ± 0.03	0.001
		Union 2	1	0.01 ± 0.02	0.5 ± 1.1	6.3 ± 13.5	99.8 ± 0.3	0.01 ± 0.01	1.000
		Union 3	1	0.01 ± 0.02	0.5 ± 1.1	6.3 ± 13.5	99.8 ± 0.3	0.01 ± 0.01	1.000
		Intersection	-	-	-	-	-	-	-
**kNN**	100%	-	12	0.37 ± 0.01	33.1 ± 1.4	41.5 ± 2.0	92.5 ± 0.6	0.28 ± 0.02	0.001
	50%	Union	10	0.38 ± 0.02	32.1 ± 1.6	46.9 ± 3.3	94.1 ± 0.8	0.30 ± 0.02	0.001
		Union 2	8	0.35 ± 0.02	29.1 ± 1.9	45.1 ± 1.8	94.3 ± 0.4	0.27 ± 0.02	0.001
		Union 3	5	0.31 ± 0.03	26.1 ± 2.9	36.9 ± 2.6	92.8 ± 0.7	0.22 ± 0.03	0.001
		Intersection	1	0.30 ± 0.07	24.8 ± 7.8	41.1 ± 5.5	93.8 ± 3.0	0.22 ± 0.05	0.001
	25%	Union	7	0.37 ± 0.03	32.3 ± 2.5	42.6 ± 3.3	93.0 ± 0.5	0.28 ± 0.03	0.001
		Union 2	3	0.38 ± 0.02	31.2 ± 2.1	47.6 ± 2.4	94.5 ± 0.4	0.30 ± 0.02	0.001
		Union 3	1	0.30 ± 0.07	24.8 ± 7.8	41.1 ± 5.5	93.8 ± 3.0	0.22 ± 0.05	0.001
		Intersection	1	0.30 ± 0.07	24.8 ± 7.8	41.1 ± 5.5	93.8 ± 3.0	0.22 ± 0.05	0.001
	10%	Union	2	0.37 ± 0.06	32.9 ± 8.2	43.6 ± 5.8	93.1 ± 1.9	0.29 ± 0.06	0.001
		Union 2	1	0.30 ± 0.07	24.8 ± 7.8	41.1 ± 5.5	93.8 ± 3.0	0.22 ± 0.05	0.001
		Union 3	1	0.30 ± 0.07	24.8 ± 7.8	41.1 ± 5.5	93.8 ± 3.0	0.22 ± 0.05	0.001
		Intersection	-	-	-	-	-	-	-
**SVM**	100%	-	12	0.36 ± 0.02	34.2 ± 2.1	37.2 ± 2.1	90.7 ± 0.7	0.26 ± 0.02	0.001
	50%	Union	10	0.37 ± 0.02	35.9 ± 2.5	38.0 ± 2.0	90.6 ± 0.4	0.27 ± 0.02	0.001
		Union 2	8	0.37 ± 0.02	35.7 ± 2.1	38.7 ± 1.8	90.9 ± 0.7	0.27 ± 0.02	0.001
		Union 3	5	0.30 ± 0.03	24.1 ± 2.6	39.7 ± 2.7	94.1 ± 0.4	0.22 ± 0.03	0.001
		Intersection	1	0.00 ± 0.00	0.0 ± 0.0	0.0 ± 0.0	1.0 ± 0.0	0.00 ± 0.00	1.000
	25%	Union	7	0.37 ± 0.02	33.8 ± 2.6	42.0 ± 2.4	92.5 ± 0.5	0.29 ± 0.03	0.001
		Union 2	3	0.29 ± 0.04	21.4 ± 3.8	45.7 ± 4.6	95.9 ± 0.4	0.22 ± 0.04	0.001
		Union 3	1	0.00 ± 0.00	0.0 ± 0.0	0.0 ± 0.0	1.0 ± 0.0	0.00 ± 0.00	1.000
		Intersection	1	0.00 ± 0.00	0.0 ± 0.0	0.0 ± 0.0	1.0 ± 0.0	0.00 ± 0.00	1.000
	10%	Union	2	0.24 ± 0.02	15.1 ± 1.4	57.6 ± 6.1	98.2 ± 0.4	0.19 ± 0.02	0.001
		Union 2	1	0.00 ± 0.00	0.0 ± 0.0	0.0 ± 0.0	1.0 ± 0.0	0.00 ± 0.00	1.000
		Union 3	1	0.00 ± 0.00	0.0 ± 0.0	0.0 ± 0.0	1.0 ± 0.0	0.00 ± 0.00	1.000
		Intersection	-	-	-	-	-	-	-

**Table A12:** Performance of the four classification models on detection fall using Early Mobility Impairment Questionnaire *data set*; Gaussian Naive Bayes (GNB); Decision Tree (DT); k-Nearest Neighbor (kNN); Support Vector Machine (SVM); *p*-value via permutation test.

	Thresh-Old	Combination Method	No. Features	F1	Recall (%)	Precision (%)	Specificity (%)	Kappa	*p*
**GNB**	100%	-	9	0.54 ± 0.03	71.3 ± 0.3	43.1 ± 0.3	84.8 ± 0.2	0.44 ± 0.00	0.001
	50%	Union	8	0.54 ± 0.00	70.2 ± 0.2	44.1 ± 0.3	85.7 ± 0.1	0.45 ± 0.00	0.001
		Union 2	5	0.54 ± 0.00	66.0 ± 0.7	45.7 ± 0.5	87.3 ± 0.2	0.45 ± 0.01	0.001
		Union 3	5	0.54 ± 0.00	66.0 ± 0.7	45.7 ± 0.5	87.3 ± 0.2	0.45 ± 0.01	0.001
		Intersection	2	0.50 ± 0.00	55.6 ± 0.7	45.7 ± 0.0	89.4 ± 0.1	0.41 ± 0.00	0.001
	25%	Union	4	0.54 ± 0.01	61.0 ± 1.4	48.0 ± 0.5	89.4 ± 0.2	0.45 ± 0.01	0.001
		Union 2	3	0.53 ± 0.00	59.9 ± 1.1	48.1 ± 0.3	89.6 ± 0.3	0.45 ± 0.00	0.001
		Union 3	1	0.45 ± 0.00	40.1 ± 0.0	51.5 ± 0.0	93.9 ± 0.0	0.37 ± 0.00	0.001
		Intersection	-	-	-	-	-	-	-
	10%	Union	2	0.48 ± 0.01	50.2 ± 0.8	45.3 ± 0.6	90.2 ± 0.1	0.39 ± 0.01	0.001
		Union 2	1	0.45 ± 0.00	40.1 ± 0.0	51.5 ± 0.0	93.9 ± 0.0	0.37 ± 0.00	0.001
		Union 3	1	0.45 ± 0.00	40.1 ± 0.0	51.5 ± 0.0	93.9 ± 0.0	0.37 ± 0.00	0.001
		Intersection	-	-	-	-	-	-	-
**DT**	100%	-	9	0.35 ± 0.06	27.4 ± 6.2	49.0 ± 4.4	95.5 ± 0.7	0.28 ± 0.06	0.001
	50%	Union	8	0.35 ± 0.06	27.4 ± 6.2	49.1 ± 4.5	95.5 ± 0.8	0.28 ± 0.06	0.001
		Union 2	5	0.35 ± 0.07	26.3 ± 6.6	54.9 ± 3.7	96.5 ± 0.9	0.29 ± 0.06	0.001
		Union 3	5	0.35 ± 0.07	26.3 ± 6.6	54.9 ± 3.7	96.5 ± 0.9	0.29 ± 0.06	0.001
		Intersection	2	0.02 ± 0.04	1.0 ± 2.1	5.7 ± 12.0	99.5 ± 0.8	0.01 ± 0.02	0.001
	25%	Union	4	0.32 ± 0.04	23.7 ± 3.6	48.5 ± 2.2	96.0 ± 0.4	0.25 ± 0.03	0.001
		Union 2	3	0.31 ± 0.03	22.2 ± 2.8	49.8 ± 2.7	96.4 ± 0.2	0.24 ± 0.03	0.001
		Union 3	1	0.39 ± 0.00	29.9 ± 0.6	54.6 ± 1.8	96.0 ± 0.4	0.32 ± 0.00	0.001
		Intersection	-	-	-	-	-	-	-
	10%	Union	2	0.31 ± 0.03	22.3 ± 2.5	51.4 ± 2.8	96.6 ± 0.2	0.25 ± 0.03	0.001
		Union 2	1	0.39 ± 0.00	29.9 ± 0.6	54.6 ± 1.8	96.0 ± 0.4	0.32 ± 0.00	0.001
		Union 3	1	0.39 ± 0.00	29.9 ± 0.6	54.6 ± 1.8	96.0 ± 0.4	0.32 ± 0.00	0.001
		Intersection	-	-	-	-	-	-	-
**kNN**	100%	-	9	0.35 ± 0.03	29.0 ± 2.4	44.5 ± 2.7	94.2 ± 0.4	0.27 ± 0.03	0.001
	50%	Union	8	0.36 ± 0.03	28.0 ± 2.1	49.5 ± 3.9	95.4 ± 0.5	0.29 ± 0.03	0.001
		Union 2	5	0.35 ± 0.02	27.7 ± 2.7	48.8 ± 2.4	95.3 ± 0.5	0.28 ± 0.02	0.001
		Union 3	5	0.35 ± 0.02	27.7 ± 2.7	48.8 ± 2.4	95.3 ± 0.5	0.28 ± 0.02	0.001
		Intersection	2	0.29 ± 0.06	22.9 ± 5.9	40.2 ± 3.6	94.6 ± 1.2	0.21 ± 0.05	0.001
	25%	Union	4	0.37 ± 0.03	31.3 ± 3.6	45.8 ± 4.4	94.0 ± 1.2	0.29 ± 0.03	0.001
		Union 2	3	0.39 ± 0.03	32.6 ± 4.5	48.1 ± 3.9	94.2 ± 1.4	0.31 ± 0.03	0.001
		Union 3	1	0.27 ± 0.06	19.1 ± 5.9	51.1 ± 8.2	96.7 ± 2.0	0.21 ± 0.05	0.001
		Intersection	-	-	-	-	-	-	-
	10%	Union	2	0.31 ± 0.04	24.5 ± 4.2	43.5 ± 5.0	94.8 ± 1.0	0.24 ± 0.04	0.001
		Union 2	1	0.27 ± 0.06	19.1 ± 5.9	51.1 ± 8.2	96.7 ± 2.0	0.21 ± 0.05	0.001
		Union 3	1	0.27 ± 0.06	19.1 ± 5.9	51.1 ± 8.2	96.7 ± 2.0	0.21 ± 0.05	0.001
		Intersection	-	-	-	-	-	-	-
**SVM**	100%	-	9	0.38 ± 0.03	30.4 ± 2.8	51.1 ± 3.4	95.3 ± 0.5	0.31 ± 0.03	0.001
	50%	Union	8	0.39 ± 0.03	31.4 ± 2.8	52.9 ± 2.7	95.5 ± 0.3	0.32 ± 0.03	0.001
		Union 2	5	0.37 ± 0.02	30.2 ± 2.2	49.0 ± 2.7	94.9 ± 0.4	0.30 ± 0.02	0.001
		Union 3	5	0.37 ± 0.02	30.2 ± 2.2	49.0 ± 2.7	94.9 ± 0.4	0.30 ± 0.02	0.001
		Intersection	2	0.04 ± 0.04	2.0 ± 2.1	14.5 ± 13.0	98.9 ± 0.9	0.01 ± 0.02	0.032
	25%	Union	4	0.32 ± 0.02	24.5 ± 1.8	46.2 ± 3.7	95.4 ± 0.5	0.25 ± 0.03	0.001
		Union 2	3	0.34 ± 0.03	26.2 ± 2.8	48.9 ± 2.3	95.6 ± 0.4	0.27 ± 0.03	0.001
		Union 3	1	0.38 ± 0.03	29.0 ± 2.9	54.1 ± 2.1	96.0 ± 0.4	0.31 ± 0.03	0.001
		Intersection	-	-	-	-	-	-	-
	10%	Union	2	0.39 ± 0.03	29.8 ± 3.1	54.7 ± 2.0	96.0 ± 0.2	0.32 ± 0.03	0.001
		Union 2	1	0.38 ± 0.03	29.0 ± 2.9	54.1 ± 2.1	96.0 ± 0.4	0.31 ± 0.03	0.001
		Union 3	1	0.38 ± 0.03	29.0 ± 2.9	54.1 ± 2.1	96.0 ± 0.4	0.31 ± 0.03	0.001
		Intersection	-	-	-	-	-	-	-
